# Phase Organization and Circularity in PLLA/Vitrimer Semi-interpenetrating Polymer Networks

**DOI:** 10.1021/acs.macromol.6c00678

**Published:** 2026-06-16

**Authors:** Luigi Gamberini, Alessandra Del Giudice, Lazaros Papadopoulos, Maria Cristina Righetti, Minna Hakkarainen, Luciano Galantini, Anna Liguori, Maria Letizia Focarete

**Affiliations:** 1 Department of Chemistry “Giacomo Ciamician” and INSTM UdR of Bologna, 9296 University of Bologna, via P. Gobetti 85, Bologna 40129, Italy; 2 Department of Chemistry, Sapienza University of Rome, P.le A. Moro 5, Rome 00185, Italy; 3 Department of Fibre and Polymer Technology, KTH Royal Institute of Technology, Teknikringen 58, Stockholm 10044, Sweden; 4 National Research Council, Institute for Chemical and Physical Processes (CNR-IPCF), Via G. Moruzzi 1, Pisa 56124, Italy; 5 Health Sciences & Technologies (HST) CIRI, 9296 University of Bologna, Via Tolara di Sopra 41/E, Ozzano Emilia 40064, Italy

## Abstract

We investigated, for the first time, the properties of semi-interpenetrating polymer networks (semi-IPNs) formed by curing a vitrimeric biobased covalent adaptable network (CAN) resin, labeled DOM-MVL, in the presence of PLLA. Three semi-IPNs with PLLA:DOM-MVL weight ratios of 80:20, 65:35, and 50:50 were developed. The 80:20 and 65:35 semi-IPNs exhibited a single glass transition temperature (*T*_g_), whose value was between those of pure CAN (31 °C) and pure PLLA (60 °C). In contrast, the 50:50 semi-IPN exhibited a broad *T*_g_, with a midpoint at 28 °C, attributed to the presence of uncured resin acting as a plasticizer in the PLLA phase. In the 65:35 semi-IPN, the presence of the CAN phase significantly inhibited PLLA crystallization. Conversely, the 50:50 sample exhibited a wider crystallization window and enhanced crystallization potential of the PLLA phase consistent with the presence of residual resin acting as a plasticizer. Scanning electron microscopy, conducted after PLLA removal, revealed a porous network with voids corresponding to previously PLLA crystalline domains. All semi-IPNs were mechanically reprocessed via hot pressing, and the feasibility of separating and recovering both components was demonstrated.

## Graphical abstract

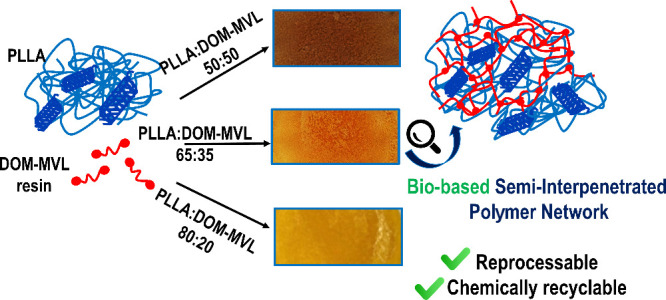

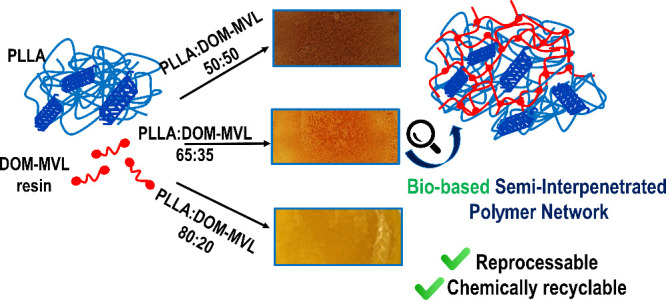

## Introduction

In recent years, the growing demand for sustainable polymers has driven the interest toward biobased thermoplastics and covalent adaptable networks (CANs). In this frame, multicomponent systems combining thermoplastics and vitrimers might represent a promising strategy to decouple material performance from end-of-life limitations, enabling more sustainable solutions for complex plastic systems. Among the thermoplastics, poly­(lactic acid) (PLA) is a highly promising material^3^ in light of its good processability,^4^ relatively low cost,^5,6^ biobased origin, biodegradability, and reprocessability.^7^

PLA exists in various stereostructures as a result of the two enantiomeric isomers (d and l) of lactic acid. Enantiomerically pure PLA, poly­(l-lactic acid) (PLLA), results in a semicrystalline polymer, characterized by an amorphous state with a glass transition temperature of approximately 60 °C and a crystalline phase presenting a melting temperature of around 175 °C.^8^ Semicrystalline PLLA exhibits high modulus and strength but also high brittleness. Modifications of PLA are often required to tailor its mechanical properties, particularly its inherent brittleness, to meet the specific demands of targeted applications. The most adopted strategies generally exploit the use of plasticizing additives^9–11^ and blending with other polymers.^10^ However, when low-molecular-weight additives are used, they can migrate from the material to the surface, causing the material to regain the inherent brittleness of neat PLA.^12–15^ The blending of PLA with oligoesters, deriving from the transesterification and esterification reactions of tributyl citrate and diethyl bis­(hydroxymethyl)­malonate, has been demonstrated to create more stable systems, showing reduced migration rates as a consequence of the increased molecular weight.^15^ An alternative approach to circumvent the migration issues involves blending PLA with other polymers.

The blending of PLLA with various fossil-based, biobased, and biodegradable polymers has been widely documented in the literature.^16,17^ Among them, blending poly­(methyl methacrylate) (PMMA) with PLLA has shown promise for automotive applications, particularly when combined with commercially available impact modifiers.^18^ This can be ascribed to the fact that PMMA restricts the crystallization ability of PLLA.^19^ This aspect, combined with the good miscibility of the two polymers during high-temperature processing,^20^ makes these blends attractive. In addition to blending approaches, semi-interpenetrating polymer networks (semi-IPNs) have been explored as an effective strategy to compatibilize immiscible polymer systems and to integrate distinct material properties. In semi-IPNs, one polymer component forms a cross-linked network that physically entangles with a second linear or branched polymer, leading to enhanced interpenetration compared to simple blends. A representative example includes semi-IPNs based on PLA and poly­(ε-caprolactone) (PCL), obtained by the reaction of methylenediphenyl 4,4′-diisocyanate with hydroxy-terminated four-arm star-shaped ε-caprolactone oligomers in the presence of PLA. Compared to the corresponding blends, these semi-IPNs exhibited reduced phase separation and improved mechanical performance.^21^ Despite these advantages, the fossil-based nature of PCL limits the sustainability of such semi-IPN systems.

To address this issue, we propose the development of innovative semi-IPNs composed of PLLA and a biobased CAN, showing a vitrimeric behavior. CANs are cross-linked networks incorporating reversible bonds that can be activated through specific stimuli, making them mechanically reprocessable and chemically recyclable.^22–29^ The CAN resin used in this study is synthesized from vanillin, a biobased molecule derived from lignin, properly functionalized to incorporate imine bonds, providing dynamic covalent adaptability, and vinyl groups for the curing into a network. The thermoplastic nature of PLLA, the presence of dynamic covalent bonds in the CAN, and the absence of covalent linkages between the two components are expected to make the materials mechanically reprocessable and furthermore allow for the separate recovery of PLLA and CAN components at the end of service life.

While some efforts have been made in the development of PLA-based CANs through the chemical modification of the thermoplastic PLLA polymer,^30,31^ no PLLA/CAN semi-IPNs have been reported so far. Given the novelty of the systems, the primary aim of this work is to elucidate the organization of the two phases. In the context of a circular economy, the mechanical reprocessing of semi-IPNs and the possibility to separate the two components at the end of service were investigated. After the separation, PLLA could be used for the fabrication of new films, while CAN could be chemically recycled.

## Materials and Methods

### Materials

Poly­(l-lactic acid) (PLLA, Lacea H.100-E, 1.5% d-configuration, 0.14% residual lactide, Mw = 8.40 × 10^4^ g/mol, PDI = 1.7) was purchased from Mitsui Fine Chemicals. Vanillin (VL, 99%), methacrylic anhydride (MAA, 94%), 2,2′-(ethylenedioxy)­bis­(ethylamine) (DOM 98%), 2-hydroxy-4′-(2-hydroxyethoxy)-2-methylpropiophenone (Irgacure 2959, 98%), 4-dimethylaminopyridine (DMAP, 99%), sodium hydrogen carbonate (for analysis), sodium hydroxide (NaOH, 98%), sodium sulfate (Na_2_SO_4_, ≥99%), and dichloromethane (DCM, ≥99%) were purchased from Sigma-Aldrich and used without any additional purification.

### Methods

#### Synthesis of DOM-MVL

The CAN resin, DOM-MVL, was synthesized according to a double-step procedure consisting of the methacrylation of VL to produce methacrylated vanillin (MVL) and the subsequent Schiff-base reaction between MVL and DOM. MVL was obtained according to the previously reported procedure.^27^ Briefly, VL (30.00 g, 197.17 mmol) was mixed with MAA (30.36 g, 197.00 mmol) and DMAP (0.17 g, 1.39 mmol) in a 250 mL round-bottom flask and kept under stirring at 60 °C for 24 h. The product of each reaction was diluted with DCM and consequently washed with a saturated aqueous solution of sodium hydrogen carbonate, 0.5 M NaOH, 1 M NaOH, and distilled water. The organic phase was dried over Na_2_SO_4_, concentrated at reduced pressure, and dried under a vacuum at room temperature for 2 days, yielding MVL as a white powder (81% reaction yield). The DOM-MVL resin was obtained by subjecting the MVL to a Schiff-base reaction with DOM.^32^ In brief, MVL (4.05 g, 18.40 mmol) and DOM (1.78 g, 12.00 mmol) were placed in a 250 mL round-bottom flask and dissolved in 60 mL of DCM. The reaction was carried out at room temperature for 5 h under stirring. Afterward, the reaction mixture was subjected to the same washing and drying procedures as described for methacrylation, yielding DOM-MVL as a pale-yellow oil (96% reaction yield).

#### Preparation of Semi-IPNs

Semi-IPNs were produced through the solvent casting of PLLA:DOM-MVL solutions in DCM followed by the curing of the resin via UV light and thermal treatment. Three PLLA:DOM-MVL weight ratios were used for semi-IPNs preparation, i.e., 80:20, 65:35, and 50:50. PLLA and DOM-MVL were separately dissolved in DCM at the concentrations reported in Table 1. The PLLA solution was then added dropwise to the DOM-MVL solution, and Irgacure 2959 was finally incorporated with a concentration of 5% w/w with respect to the weight of DOM-MVL. The resulting solution was gently poured into a circular silicone mold (5 cm inner diameter) and left to dry under ambient conditions for 24 h to allow solvent evaporation. The films were cured using a Thorlabs Solis 365-C UV lamp (365 nm) operating with a light intensity of 100 W/m^2^ for 1 h (30 min/side) and postcured through a thermal treatment at 130 °C for 90 min in a vacuum oven.

**Table 1:** Concentrations of PLLA and DOM-MVL Solutions Used for the Preparation of Semi-IPNs

sample labels	weight PLLA [g]/volume DCM [mL]	weight DOM-MVL [g]/volume DCM [mL]
PLLA	1.38/27.80	
DOM-MVL CAN		1.75/2.50
PLLA:DOM-MVL 80:20	1.10/25.80	0.28/2.00
PLLA:DOM-MVL 65:35	0.90/25.80	0.48/2.00
PLLA:DOM-MVL 50:50	0.69/25.80	0.69/2.00

As a comparison, the PLLA film and DOM-MVL CAN were also produced. The PLLA film was obtained by solvent casting a 5% w/v PLLA solution in DCM for 24 h. The CAN was fabricated by dissolving DOM-MVL at 70% w/v in DCM and adding 5% w/w of Irgacure 2959. The solution was cast and cured, after solvent evaporation, under identical UV and thermal conditions as those used for the semi-IPNs.

All of the samples were dried under a vacuum until a constant weight was reached and were labeled as per Table 1.

#### Characterizations

The chemical structure of MVL and DOM-MVL was investigated through ^1^H NMR spectroscopy performed on an Avance 600 (Bruker) spectrometer (600 MHz) equipped with a 5.0 mm multinuclear observe probe with a *Z*-gradient. CDCl_3_ was used as the solvent and as the internal standard for calibrating the chemical shift. Chemical structures of all the monomers and solid samples were characterized through ATR-FTIR using an ALPHA II PLATINUM-ATR (Bruker Corporation). All of the spectra were recorded in the wavenumber range of 4000–400 cm^–1^ using 48 scans at a resolution of 4 cm^–1^. Thermogravimetry analysis (TGA) was performed by a TGA Q500 (TA Instruments). Samples with a weight between 5 and 10 mg were subjected to a heating scan from 30 to 700 °C with a heating rate of 10 °C/min in a N_2_ atmosphere. Differential scanning calorimetry (DSC) analysis of the samples was performed using a DSC Q2000 instrument (TA Instruments) equipped with a refrigerated cooling system (RCS) in a N_2_ atmosphere. The sample (5–10 mg) was sealed into an aluminum crucible and subjected to three heating scans from −90 to 190 °C with a heating rate of 20 °C/min. A quench and a controlled cooling (cooling ramp 10 °C/min) were performed after the first and second heating scan, respectively. *T*_g_ was taken as the half-height of the glass transition heat capacity step. DSC measurements were performed in triplicate on cured semi-IPNs obtained from three different films for each composition.

Melting and crystallization enthalpies, denoted as Δ*H*_m_ and Δ*H*_c_, were determined from DSC measurements. These values were also used to calculate the normalized enthalpies based on the PLLA weight fraction in each sample according to the following equations:

$$\Delta H_{\mathrm{mPLLA}}=\frac{\Delta H_{m}}{w_{\mathrm{PLLA}}}$$ 1

$$\Delta H_{\mathrm{cPLLA}}=\frac{\Delta H_{c}}{w_{\mathrm{PLLA}}}$$ 2

where Δ*H*_mPLLA_ and Δ*H*_cPLLA_ are the normalized melting and crystallization enthalpies based on the PLLA weight fraction in the sample, Δ*H*_m_ and Δ*H*_c_ are the measured enthalpies, amd *w*_PLLA_ is the PLLA weight fraction in the sample.

Dynamic mechanical thermal analysis (DMTA) was performed on all the samples by using a DMA Q800 (TA Instruments). The measurements were performed in a multifrequency strain mode with a static force of 0.01 N at a frequency of 1.0 Hz. The deflection amplitude of oscillation and Poisson’s ratio were set at 4.0 μm and 0.44, respectively. The samples were equilibrated at 25 °C for 2 min and tested from 25 to 135 °C at 1 Hz with a 2 °C/min heating rate. The glass transition temperature was considered as the maximum of the tan δ curve.

The development of the PLLA crystal phase has been investigated on the PLLA film and all the semi-IPNs by means of an optical microscope (Zeiss Axiocam 208) equipped with the software ZEN 3.6 and coupled with a heating optical stage (LINKAM T96). All the samples were heated from RT to 210 °C with a 50 °C/min heating ramp. Once the melting of PLLA crystals in the PLLA film and in the semi-IPNs was completed, samples were quenched to 115 °C and kept at this temperature for 15 min before collecting the images.

Structural differences among the semi-IPNs with different DOM-MVL CAN contents at the nano- and mesoscale, in addition to the detection of crystallinity of PLLA, were highlighted with small- and wide-angle X-ray scattering (SAXS and WAXS). The measurements were performed with a Xeuss 2.0 Q-Xoom system (Xenocs SAS, Grenoble, France) equipped with a microfocus Genix 3D X-ray source with a Cu anode (λ = 0.1542 nm), a two-dimensional detector that can be placed at a variable distance from the sample, and an additional WAXS detector in a fixed position (Pilatus3 R, Dectris Ltd., Baden, Switzerland). The beam size was defined to be 0.5 × 0.5 mm through the two-pinhole collimation system equipped with “scatterless” slits. Calibration of the scattering vector *q* range (*q* = 4πsin θ/λ with 2θ the scattering angle and λ the photon wavelength) was performed using silver behenate for the SAXS and Al_2_O_3_ for the WAXS detector, respectively. Measurements with four sample-to-detector distances were performed so that the overall explored *q* region was 0.04 nm^–1^ < *q* < 33 nm^–1^. The circular samples cut to a diameter of 6 mm were fixed onto a sample holder for solids and measured in the instrument sample chamber at reduced pressure (∼0.4 mbar) at room temperature (22–24 °C). The two-dimensional scattering patterns collected with a total acquisition time of 30 min per configuration were corrected by subtracting the dark counts, and then masked, azimuthally averaged, and normalized for transmitted beam intensity, exposure time, and subtended solid angle per pixel using the software Foxtrot (Soleil, Orsay, France). The one-dimensional intensity vs *q* profiles were then background-corrected by subtracting the data of the empty chamber and divided by the average sample thickness to obtain the intensity in units of macroscopic scattering cross section (cm^–1^). The different angular ranges were merged using the SAXS utilities tool.^33^ The values of sample thickness measured by Mitutoyo Quantumike IP65 (±0.001 mm) and used for normalization were 0.0877 cm (PLLA), 0.0588 cm (DOM-MVL CAN), 0.0846 cm (PLLA:DOM-MVL 80:20), 0.0628 cm (PLLA:DOM-MVL 65:35), and 0.0569 cm (PLLA:DOM-MVL 50:50). Additional measurements were conducted as a function of temperature, mounting samples onto a Peltier-controlled sample holder (Xenocs). Starting from 15 °C, the temperature was set stepwise at 30, 45, 60, 75, and 90 °C, waiting for 6 h per step during data collection, and finally back to 20 °C within 0.5 h (quenching). Calculation of the one-dimensional correlation function was performed with SasView 6.1.2 (sasview.org) extrapolating the data via the Guinier (*q* < 0.055 nm^–1^) and Porod (*q* > 1.25 nm^–1^) approximations. SAXS and WAXS measurements were repeated on two different film aliquots for each composition and checked for consistency.

Solubility tests in DCM were performed to collect information on the solvent resistance properties of the semi-IPNs. Around 30 mg of each semi-IPN and DOM-MVL CAN was immersed in 5 mL of DCM for 72 h. Afterward, DCM was removed, and the samples were washed with fresh DCM and allowed to dry first in air for 24 h and then under a vacuum until a constant weight was reached. The collected solid fraction, referred to as residue, was calculated according to eq 3:

$$\mathrm{residue}\%=\frac{w_{f}}{w_{i}}\%$$ 3

where *w*_f_ is the weight of the solid samples collected after immersion in DCM and drying and *w*_i_ is the weight of the sample before the immersion in DCM. Tests were repeated on three different samples for each semi-IPN composition.

In addition, extraction experiments were carried out to quantitatively remove all soluble components and to get insights into the capability of DOM-MVL to cure in the presence of semicrystalline PLLA. In this case, three different samples for each semi-IPN composition were immersed in DCM under mechanical stirring (200 rpm) for 72 h. After extraction, the solid residues were recovered and dried to constant weight, while the DCM solutions were solvent-cast and further analyzed by TGA to estimate the fraction of unreacted or oligomeric DOM-MVL species in the extracts.

The morphology of all semi-IPN and of DOM-MVL CAN before and after the solubility tests was investigated through scanning electron microscopy (SEM, Leica Cambridge Stereoscan 360) with an acceleration voltage of 20 kV. The samples were attached to an aluminum stub using carbon tape and sputter-coated with gold for 150 s.

Stress–strain tests were carried out using a Linkam MFS350 microtensile stage equipped with a 200 N load cell, controlled by the accompanying Link software. Rectangular-shaped samples (gauge length = 15 mm, width = 10–15 mm, and thickness = 0.05–0.2 mm) were strained at 0.5%·s^–1^. For each material, at least three samples of each type were tested.

#### Mechanical Reprocessing

A 0.8 g portion of each sample was ground, transferred to a circular mold (diameter 2 cm), and hot-pressed at 200 °C and 20 bar for 5 min.

#### Recovery of the Two Components and Chemical Recycling of CAN

One gram of PLLA:DOM-MVL 65:35 was immersed in 5 mL of DCM for 72 h to induce the separation of PLLA, which was solubilized in DCM, from DOM-MVL CAN. DOM-MVL was then collected as the remaining solid residue. The DCM solution was solvent cast, and the resulting film was immersed in acetone for 1 h and then dried in a vacuum oven overnight at 130 °C. The solid component was then subjected to the chemical recycling procedure.^28,34^ Briefly, 0.11 g of solid material was immersed in 2 mL of DOM for 5 h at 40 °C. The resulting oligomeric product was collected by inducing its precipitation in water followed by suction filtration. After drying, the amine-end-group-functionalized powder was mixed with fresh MVL with a 2:1 w/w ratio, and Irgacure 2959 was added with a 5% w/w concentration. The resulting powder was hot-pressed at 120 °C and 20 bar for 2 min.

## Results and Discussion

### Semi-IPN Preparation

The chemical structure of MVL and DOM-MVL resin was validated through ^1^H NMR (Figure S1) and ATR-FTIR (Figure S2) analysis. From the ^1^H NMR analysis, the successful synthesis of DOM-MVL was confirmed by the signals at δ6.38 and δ5.79 ppm corresponding to the vinyl functions and the signal at δ8.26 ppm ascribed to the imine group. From the ATR-FTIR analysis, the bands at 950 and 1740 cm^–1^, absent in VL, appear in MVL, confirming the introduction of vinyl groups through the formation of ester functionalities during the methacrylation reaction. The shift from 1695 cm^–1^, characteristic of aldehyde groups in MVL, to 1650 cm^–1^ in DOM-MVL indicates the successful formation of imine bonds through the Schiff-base reaction.

The methodology used for the preparation of semi-IPNs is illustrated in Figure 1a. Solutions of PLLA and DOM-MVL resin were separately prepared at the concentrations listed in Table 1 and subsequently mixed prior to the addition of the photoinitiator. The resulting solution was solvent-cast, and after the solvent evaporation, UV curing was performed followed by a thermal post-treatment. PLLA:DOM-MVL compositions (80:20, 65:35, and 50:50 w/w %) were selected to progressively increase the content of the vitrimeric CAN phase and systematically investigate its effect on phase organization, miscibility, and crystallization behavior. A DOM-MVL-rich formulation (e.g., PLLA:DOM-MVL 20:80 w/w %) was preliminarily explored but excluded from the study due to the high amount of uncured resin and the inability to form a stable semi-IPN structure.

**Figure 1:**
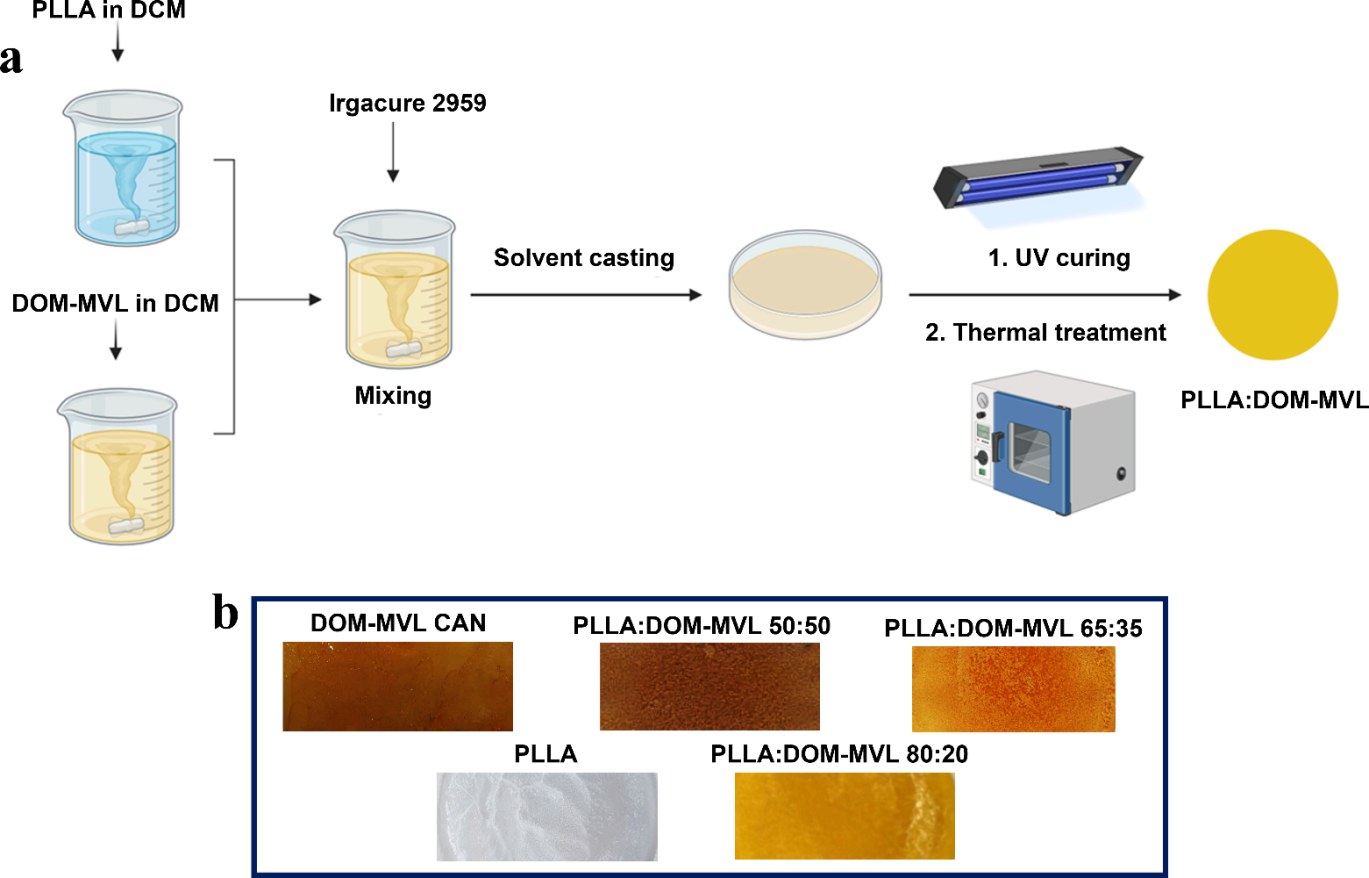
(a) Scheme of the procedure for the preparation of semi-IPN. (b) Pictures of the PLLA films, DOM-MVL CAN, and the semi-IPNs.

The curing procedure was first optimized in a preparatory study on the neat DOM-MVL resin. Increasing the UV exposure time from 15 min to 1 h improved the curing efficiency, as evidenced by the reduction of the intensity of the 950 cm^–1^ band with respect to the adjacent 870 cm^–1^ band (Figure S3a). However, the exothermic transition observed at 107 °C during the first DSC heating scan indicated that the curing of the resin remained incomplete after 1 h of UV exposure (red curve in Figure S3b). To address this, various thermal post-treatment conditions were systematically investigated by varying the temperature from 110 to 130 °C and the duration from 15 min to 1.5 h. Optimal conditions were identified as 130 °C for 1.5 h, as significant exothermic signals were no longer observed by DSC (Figure S3b and Table S1), indicating complete curing during the post-treatment step. The same thermal protocol was applied to the PLLA:DOM-MVL systems, as the UV exposure alone was insufficient to achieve complete resin curing, as evidenced by the broad exothermic signal between 80 and 140 °C in the first DSC heating scan (Figure S4).

Photographs of the resulting semi-IPNs, along with those of the PLLA film and DOM-MVL CAN, are shown in Figure 1b. The images clearly document the transition in color from white to brown as the content of DOM-MVL resin in the solvent-cast solution increases.

The incorporation of DOM-MVL and the possible interactions between PLLA and the CAN phase were investigated by ATR-FTIR spectroscopy (Figure 2a,b). The FTIR spectrum of PLLA is characterized by a strong ester carbonyl stretching band at approximately 1748 cm^–1^, along with C–O–C stretching vibrations in the 1180–1080 cm^–1^ region and aliphatic C–H stretching modes between 2995 and 2945 cm^–1^. The DOM-MVL CAN spectrum exhibits a broad absorption band around 3300 cm^–1^, attributed to absorbed moisture interacting with polar ether groups, as well as aliphatic C–H stretching bands in the 2800–2900 cm^–1^ region. An ester carbonyl stretching band is observed at ∼ 1736 cm^–1^, associated with the methacrylate-derived ester functionalities. Additional bands at approximately 1417, 1505, and 1590 cm^–1^ correspond to vibrations of methylene groups adjacent to ether moieties and to aromatic ring stretching modes, while the band at ∼1640 cm^–1^ is assigned to the aromatic–imine conjugated structure, including contributions from C=N stretching. In the semi-IPNs, the intensity of the absorption bands associated with DOM-MVL increases systematically with increasing resin content, confirming the incorporation of the CAN phase. Only a modification in the shape of the ester carbonyl stretching band is observed. Although this band remains within the same wavenumber range for PLLA and the semi-IPNs, its maximum shifts from 1748 cm^–1^ in neat PLLA and the PLLA:DOM-MVL 80:20 semi-IPN to approximately 1754 cm^–1^ in compositions with higher CAN content. This shift could be attributed to changes in the local chemical environment of the PLLA chains due to the formation of the CAN network and weak dipolar interactions between PLLA and DOM-MVL.

**Figure 2:**
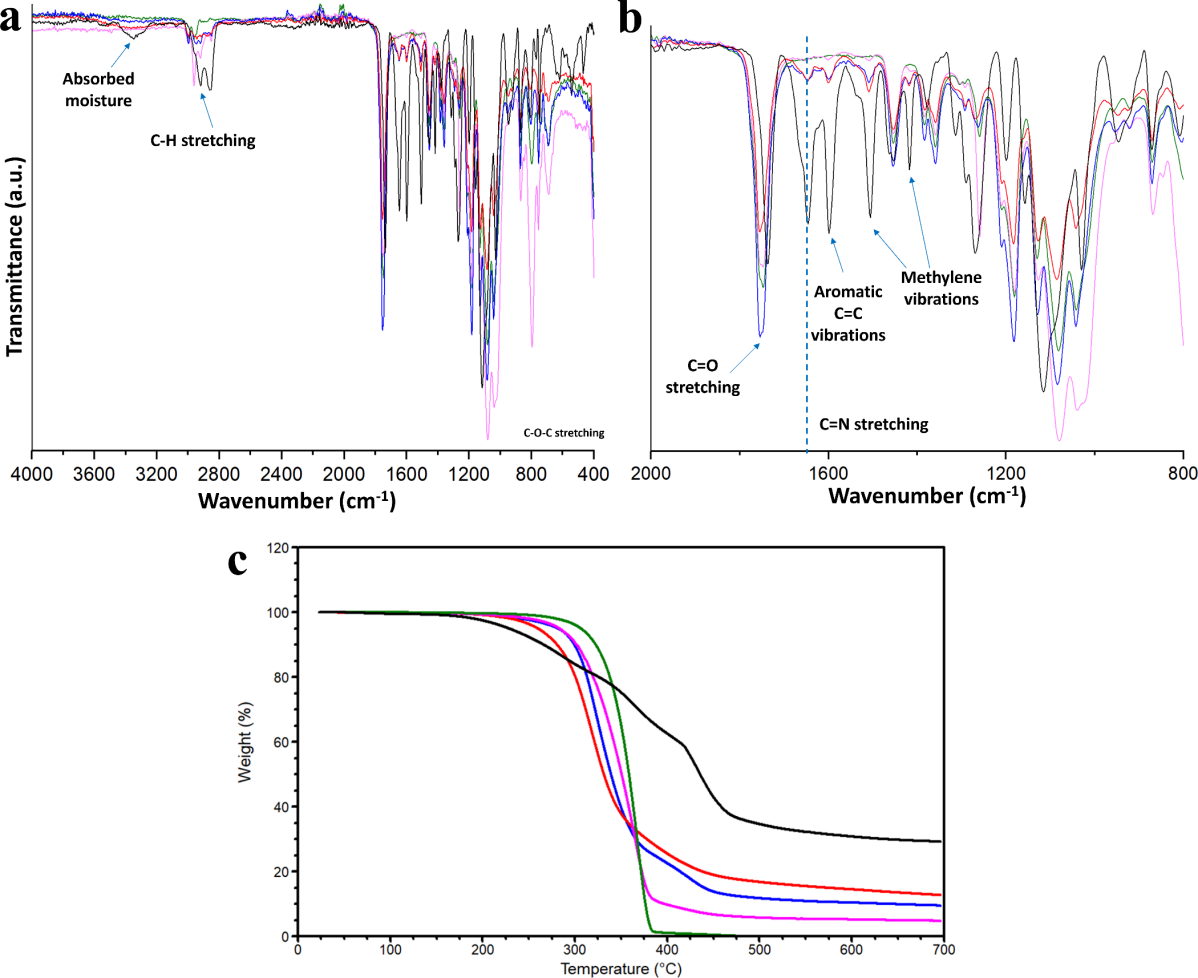
Characterization of the composition of semi-IPNs. (a) ATR-FTIR spectra over the full wavenumber range and (b) in the 2000–800 cm^–1^ region. (c) TGA curves of DOM-MVL CAN (black), PLLA:DOM-MVL 50:50 (red), PLLA:DOM-MVL 65:35 (blue), PLLA:DOM-MVL 80:20 (pink), and PLLA (green).

The actual amount of resin used for each semi-IPN was determined using TGA analysis by evaluating the residue at 700 °C (Figure 2c). Although the presence of PLLA affects the thermal degradation profile of DOM-MVL CAN, the quantification of the CAN content was based exclusively on the residual mass at high temperature, where PLLA is fully decomposed and only the aromatic-rich CAN phase is expected to contribute to the residue.^35^ Indeed, at this temperature, no residue was detected for PLLA, while a residue of 29% w/w was measured for DOM-MVL CAN prepared from 100% DOM-MVL resin. Using these values, the effective concentration of DOM-MVL present in the semi-IPNs was estimated, with the results shown in Table 2 aligning closely with the theoretical compositions. The TGA curves of the semi-IPNs indicate thermal degradation behaviors intermediate between those of PLLA and DOM-MVL CAN. All the semi-IPNs show good thermal stability, with degradation onset (*T*_deg5%_) above 200 °C and maximum degradation rate (*T*_v deg max_) in the range 317–366 °C. DOM-MVL CAN exhibited the lowest thermal stability, potentially due to the presence of irregularities in the network that could serve as initiation points for thermal degradation as well as to the presence of imine functionalities, which typically display reduced thermal resistance compared to permanent covalent bonds.

**Table 2:** Thermogravimetric Data from TGA Curves and Calculated Concentration of DOM-MVL Resin Used for Sample Preparation

sample	*T*_deg5%_ [°C]	*T*_v deg max_ [°C]	residue at 700 °C [w/w %]	calculated DOM-MVL resin concentration [w/w %]
DOM-MVL CAN	228	286, 367, 432	29	100
PLLA:DOM-MVL 50:50	264	317	13	45
PLLA:DOM-MVL 65:35	282	323	10	35
PLLA:DOM-MVL 80:20	284	366	5	17
PLLA	306	368	0	

### Confirmation of the Semi-IPN Structure and Effect of DOM-MVL CAN on PLLA Crystallization

Since there are no previous studies on combining PLLA with a CAN, the semi-IPN nature of the resulting materials was first investigated. The glass transition of the systems was analyzed through DSC and DMTA to gain insights into their structural characteristics and phase organization.^36–38^ The first calorimetric heating scan on noncured PLLA:DOM-MVL systems revealed two distinct glass transitions, as shown in Figure 3a,b and in Table S2. The first *T*_g_, between −29 and −25 °C, corresponds to the DOM-MVL resin, while the second *T*_g_, in the range 45–48 °C, is slightly lower than that of pure PLLA, probably due to the plasticizing action of a portion of CAN resin on PLLA macromolecules. For the PLLA:DOM-MVL = 80:20 system, the first *T*_g_ was challenging to detect due the low concentration of the resin. The observation of two distinct *T*_g_ documents, in agreement with FTIR analysis results, the immiscibility of the PLLA and DOM-MVL resin.^20,38,39^

**Figure 3:**
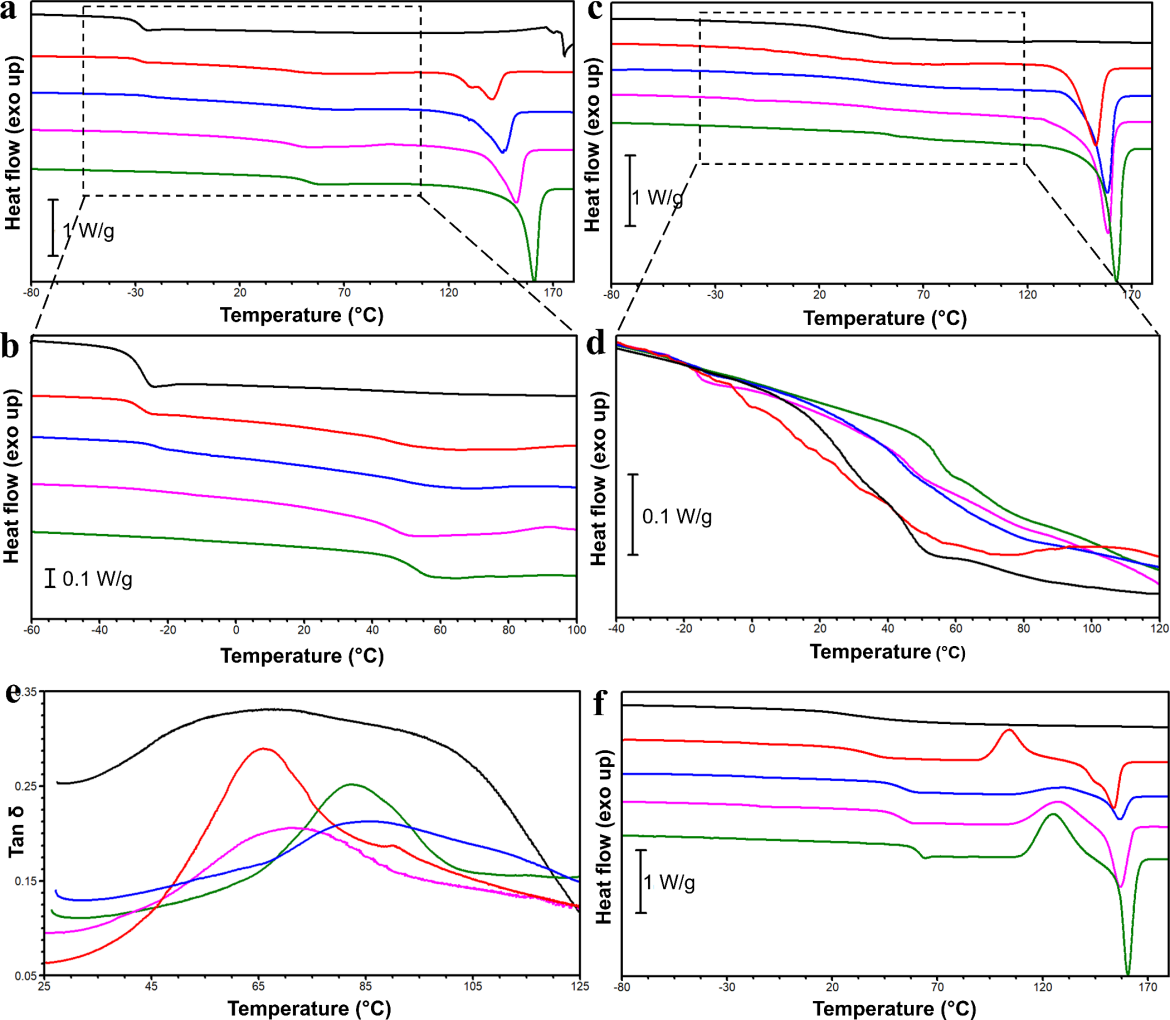
DSC and DMTA analysis. (a) First calorimetric heating scan and (b) zoomed-in image of the temperature range between −60 and 100 °C of samples not subjected to the curing procedure. (c) First calorimetric heating scan and (d) zoomed-in image of the temperature range between −40 and 120 °C of samples after the curing procedure. (e) Tan δ from DMTA analysis of samples after the curing procedure and (f) second calorimetric heating scan. DOM-MVL resin or CAN (black), PLLA:DOM-MVL 50:50 (red), PLLA:DOM-MVL 65:35 (blue), PLLA:DOM-MVL 80:20 (pink), and (green).

After the curing procedure, a single *T*_g_ was detected for all the systems (Figure 3c,d and Table 3, first heating scan). The *T*_g_ of pure DOM-MVL after curing appears much higher than that of the uncured resin, which is consistent with the formation of the cured network. For PLLA:DOM-MVL = 80:20 and PLLA:DOM-MVL = 65:35, the glass transition includes the contributions of both PLLA and DOM-MVL CAN, with the midpoint of this transition lying between the *T*_g_ values of the two pure phases, consistent with the formation of semi-IPNs,^40,41^ as detailed in Table 3. Since the *T*_g_’s of PLLA and DOM-MVL are not far from each other, the glass transitions of the semi-IPNs could result from the superposition of the two separate *T*_g_’s. PLLA:DOM-MVL = 50:50 showed a broader glass transition with the half-height of its heat capacity step at 28 °C. This value lies outside the range between the *T*_g_ of DOM-MVL CAN (31 °C) and the one of PLLA (60 °C). This behavior is consistent with the presence of a high concentration of uncured or oligomeric resin species, which likely act as plasticizers within the PLLA phase. Indeed, as schematized in Figure S5, it is hypothesized that the formation of cocontinuous PLLA and CAN phases characterizes the semi-IPNs 80:20, 65:35, and 50:50, with the peculiarity that for the highest DOM-MVL amount (semi-IPNs 50:50), a higher concentration of isolated uncured resin monomers can remain trapped within the PLLA phase (Figure S5). These residual DOM-MVL monomers may contribute to a plasticizing effect with the PLLA matrix. This hypothesis is further supported by the appearance of an exothermic peak at 113 °C in the DSC thermogram of PLLA:DOM-MVL = 50:50 (Figure 3d), attributed to additional curing of the resin component and indicating incomplete curing during the thermal post-treatment. Conversely, at the lowest DOM-MVL amount (semi-IPNs 80:20), due to the prevalence of the PLLA phase, the more dispersed DOM-MVL monomers cannot react totally, remaining as inclusions partially segregated in the thermoplastic matrix.

**Table 3:** DSC Data from the First and the Second Heating Scan for Cured Systems

	first heating scan				second heating scan (after quench)						
sample	*T*_g_ [°C]	*T*_m_ [°C]	Δ*H*_m_ [J/g]	Δ*H*_mPLLA_ [J/g]	*T*_g_ [°C]	*T*_c_ [°C]	Δ*H*_c_ [J/g]	Δ*H*_cPLLA_ [J/g]	*T*_m_ [°C]	Δ*H*_m_ [J/g]	Δ*H*_mPLLA_ [J/g]
DOM-MVL CAN	31 ± 1				32 ± 1						
PLLA:DOM-MVL 50:50	28 ± 3	152 ± 1	36 ± 3	72 ± 3	34 ± 3	104 ± 3	24 ± 3	48 ± 3	154 ± 1	25 ± 3	50 ± 3
PLLA:DOM-MVL 65:35	49 ± 4	160 ± 1	42 ± 3	65 ± 3	55 ± 2	131 ± 2	13 ± 2	20 ± 2	158 ± 2	15 ± 2	23 ± 2
PLLA:DOM-MVL 80:20	48 ± 2	160 ± 1	50 ± 2	63 ± 2	51 ± 1	124 ± 1	31 ± 1	39 ± 1	158 ± 1	32 ± 2	40 ± 2
PLLA	60 ± 2	164 ± 2	57 ± 3	57 ± 3	61 ± 1	125 ± 1	40 ± 2	40 ± 2	162 ± 2	40 ± 2	40 ± 2

Unlike the curing of plain DOM-MVL CAN, curing in the semi-IPNs is intrinsically more challenging due to the presence of the semicrystalline PLLA phase grown upon solvent casting, which can result in the presence of residual reactive resin detectable during the first heating scan in DSC after curing. Indeed, the DSC curves before and after curing (Figure 3a,c) prove that the PLLA crystalline structure grows during the solvent casting step and in addition during the thermal treatment at 130 °C for 90 min, as attested by the Δ*H*_m_ value that increases from 47 (Figure 3a) to 57 J/g (Figure 3c). This means that the DOM-MVL network undoubtedly develops in the presence of the PLLA crystals, which could hinder the complete cross-linking.

Additionally, the *T*_g_ of PLLA:DOM-MVL = 80:20 appears slightly lower than that of PLLA:DOM-MVL = 65:35 (see Table 3), which may be associated with the presence of nonreacted resin, trapped as dispersed inclusions within the PLLA matrix. Although the curing protocol was optimized to maximize network formation, the amount of unreacted or oligomeric material in the semi-IPNs appears to be more influenced by the composition-dependent phase organization rather than solely by the curing protocol, particularly for DOM-MVL-rich formulations.

The hypotheses on the separate organization of the two components were further investigated using complementary experimental techniques (see below). The DMTA curves, shown in Figure 3e, corroborate the presence of a single glass transition in alignment with the DSC results. The tan δ curve for DOM-MVL CAN exhibits a broad distribution across a wide range of temperatures, consistent with structural irregularities in the network.^34^ For the semi-IPNs, the PLLA:DOM-MVL = 80:20 and 65:35 compositions exhibit tan δ maxima at temperatures slightly below and coincident with the pure PLLA (82 °C), respectively. The lower maximum temperature (72 °C) observed for PLLA:DOM-MVL = 80:20 indicates the presence of a fraction of unreacted monomer, which likely acts as a plasticizer for the PLLA macromolecules. Conversely, the alignment of the tan δ peak for PLLA:DOM-MVL = 65:35 with pure PLLA suggests the highest curing degree of the resin in this formulation and/or the presence of only a minor amount of plasticizing species. For the PLLA:DOM-MVL = 50:50 system, the tan δ curve resembles that of PLLA but with a maximum shifted to a substantially lower temperature (65 °C). This shift indicates the presence of unreacted DOM-MVL resin, which markedly reduces the *T*_g_ of PLLA. Finally, the upper-temperature segment of the tan δ peak is broader for all semi-IPNs, reflecting restricted chain mobility due to the tight packing of PLLA and CAN.^37,42^ This effect is particularly pronounced for the PLLA:DOM-MVL = 65:35 system, suggesting a higher packing density of the macromolecules in this composition.

To investigate the influence of the DOM-MVL CAN phase on the crystallization behavior of PLLA in the semi-IPN systems, calorimetric heating scans were conducted following both quench cooling and controlled cooling after melting of the PLLA phase. The first heating scan after curing was excluded from the analysis to avoid potential interference of residual unreacted resin, which may cure during the DSC measurements and obscure accurate determination of the melting enthalpy as well as comparisons with neat PLLA.

Considering the similarities observed between the cycles following controlled and quench cooling, the heating scan after quench was selected for comparison of the semi-IPNs with pure PLLA, as shown in Figure 3f. After the melting of PLLA’s crystalline phase during the first heating scan, PLLA and all the semi-IPNs were completely amorphous, as evidenced by the equivalence of crystallization and melting enthalpies (Table 3, second heating cycle). The additional curing occurring in the semi-IPNs during the first heating scan leads to a further growth of the DOM-MVL network, as also attested by the higher *T*_g_ values detected during the second heating scan.

After complete melting, the PLLA crystallinity that develops during the second heating scan (Figure 3f) is lower than that detected during the first heating scan after curing (Figure 3c) due to the lower time allowed for crystallization upon heating in comparison with the entire preparatory procedure of the samples (i.e., solvent casting and thermal treatment at 130 °C). For PLLA:DOM-MVL = 80:20, the presence of CAN did not hinder PLLA’s ability to develop a crystal phase, with Δ*H*_mPLLA_ coincident with the melting enthalpy of pure PLLA (Table 3). Conversely, for the semi-IPN PLLA:DOM-MVL = 65:35, the CAN phase significantly inhibited the crystallization of PLLA. In this system, Δ*H*_mPLLA_ was only 58% of the melting enthalpy of pure PLLA (Table 3). These results suggest that as the amount of DOM-MVL CAN phase is rather low in PLLA:DOM-MVL = 80:20, no significant effects are observed on PLLA’s potential to crystallize. However, increasing the DOM-MVL CAN content from 20% to 35% w/w significantly hinders the crystallization of PLLA, highlighting the more interpenetrating morphology of the material and the physical interconnection between the two phases, attesting the confinement effect exerted by the DOM-MVL network on the PLLA phase.^43^

In contrast to this trend, PLLA:DOM-MVL = 50:50 displayed a Δ*H*_mPLLA_ that was 25% higher than the expected value (Table 3). This outcome is likely related to the lower *T*_g_ observed for this sample and is consistent with the presence of noncured resin or oligomeric cross-linked structures in the PLLA phase, which may contribute to the plasticization effect in the semi-IPN. The resulting decrease in *T*_g_ extends the crystallization window to a broader temperature range, facilitating crystallization compared to neat PLLA (Figure 3e and Table 3).

In conclusion, the curing process of DOM-MVL appears dependent on the presence of the PLLA crystal phase, which slightly hinders the complete cross-linking, whereas PLLA crystallization turns out to be significantly affected by the confinement imposed by the DOM-MVL network, which can lead to a reduction in PLLA crystallinity. Conversely, a high amount of noncured DOM-MVL, with its plasticizing action, favors the PLLA crystallization.

To quantify the unreacted resin or oligomeric structures, acting as plasticizers in the semi-IPNs, DCM extraction experiments were performed under mechanical stirring (200 rpm, 72 h) to maximize the removal of all soluble species, including PLLA chains and unreacted or oligomeric DOM-MVL structures. After extraction, the DCM solution was solvent-casted, and the extracts were analyzed by ^1^H NMR and TGA. From the ^1^H NMR analysis (Figure S6), in all the spectra, characteristic signals of both PLLA and DOM-MVL species were detected, confirming that the extracted fraction consists of a mixture of PLLA and uncured or oligomeric resin species. In agreement with the DSC, DMTA, and extraction results, the PLLA:DOM-MVL 65:35 system exhibited the lowest relative amount of DOM-MVL-related signals with respect to PLLA, indicating the lowest concentration of soluble resin species among the investigated compositions. Additionally, signals attributable to both imine and aldehyde functionalities were observed in the DOM-MVL-related region, suggesting partial imine bond cleavage occurring in solution, possibly promoted by the acidic terminal groups of the PLLA macromolecules.

TGA was performed to estimate the fraction of unreacted or oligomeric DOM-MVL species in the extracts, taking into account that these materials complete their curing during the measurement. The analysis of the curves, shown in Figure S7 and Table S3, reveals that the extracts from PLLA:DOM-MVL 65:35 semi-IPN contains the lowest fraction of unreacted or partially reacted resin (≈18 wt %), while a significantly higher fraction is extracted from the 50:50 composition (≈40 wt %). Starting from this information, the amount of unreacted resin in the semi-IPN was calculated using PLLA as an internal reference by applying a simple mass proportion:

$$\frac{w\;{\%}_{\mathrm{resin},\mathrm{extract}}}{w\;{\%}_{\mathrm{PLLA},\mathrm{extract}}}=\frac{w\;{\%}_{\mathrm{unreacted}\mathrm{resin},\mathrm{semi}-\mathrm{IPN}}}{w\;{\%}_{\mathrm{PLLA},\mathrm{semi}-\mathrm{IPN}}}$$ 4

Given that PLLA:DOM-MVL 80:20 contains 17 wt % of unreacted resin or soluble oligomeric structures, the semi-IPN 65:35 then contains 14 wt % and the 50:50 system contains 33 wt % of unreacted resin. These results are consistent with DSC data, where the 65:35 system shows the strongest inhibition of PLLA crystallization due to a lower percentage of uncured resin and a more extensive phase interpenetration, whereas the higher content of soluble species in the 80:20 and 50:50 systems correlates with a reduced *T*_g_ and enhanced crystallization potential.

The ability of the DOM-MVL CAN phase to affect the crystallization of PLLA from melt was further investigated using a polarized optical microscope (Figure 4). When subjected to an isothermal temperature of 115 °C for 15 min, neat PLLA exhibited near-complete crystallization, forming a high density of spherulites (Figure 4a). This observation is consistent with earlier studies, which reported similar spherulitic morphologies for PLLA crystallized under these conditions.^44^ In contrast, the semi-IPNs (Figure 4b–d) displayed spherulites with irregular morphologies compared to those of neat PLLA, probably due to the presence of DOM-MVL CAN. The lower number of spherulites that are displayed by the sample PLLA:DOM-MVL 80:20 can be explained by considering the lower size of the PLLA regions in the presence of the DOM-MVL network, which can slow down and hinder the nucleation step. As a consequence, the lower number of nuclei can lead to larger spherulites after sufficiently long crystallization time. The progressively decreasing dimensions of the PLLA regions with increasing DOM-MVL percentage necessarily induce reduction in the PLLA crystal size of PLLA:DOM-MVL 65:35 and 50:50, as observable in Figure 4c,d. Additionally, the presence of significant amorphous regions (recognizable by the extended continuous dark areas), ascribed to the DOM-MVL CAN phase, in all the semi-IPN samples was clearly evident, documenting the coexistence of amorphous and semicrystalline regions.

**Figure 4:**
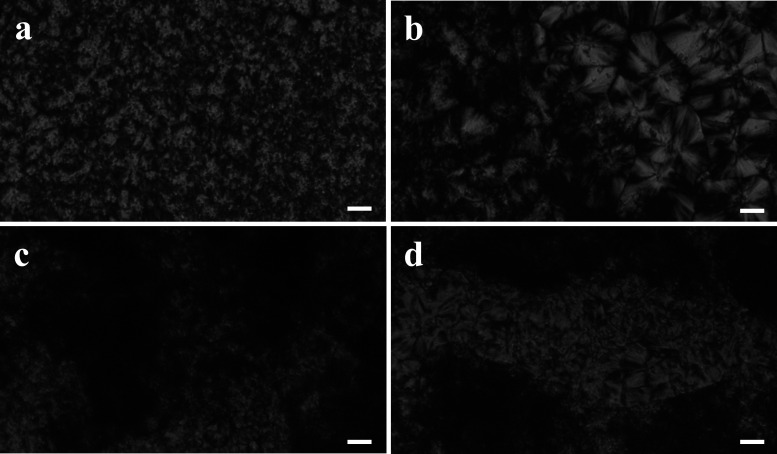
Optical microscopy of (a) PLLA, (b) PLLA:DOM-MVL 80:20, (c) PLLA:DOM-MVL 65:35, and (d) PLLA:DOM-MVL 50:50 isothermally crystallized at 115 °C for 15 min from melt. Scale bar: 20 μm.

To further investigate the organization of the two phases, SAXS and WAXS characterizations were performed.

### Structural Organization of the Two Phases at the Nanoscale

The SAXS and WAXS characterization of the semi-IPNs suggests a possible structural organization at the nanoscale (Figure 5). The PLLA-containing samples show the features of semicrystalline polymers, with high scattering signal in the low *q* (<1 nm^–1^) due to the contrast between amorphous and crystalline nanometer-sized domains with different electron density and crystalline peaks of ordered atomic packing in the wide angle (*q* > 5 nm^–1^) (Figure 5a).

**Figure 5:**
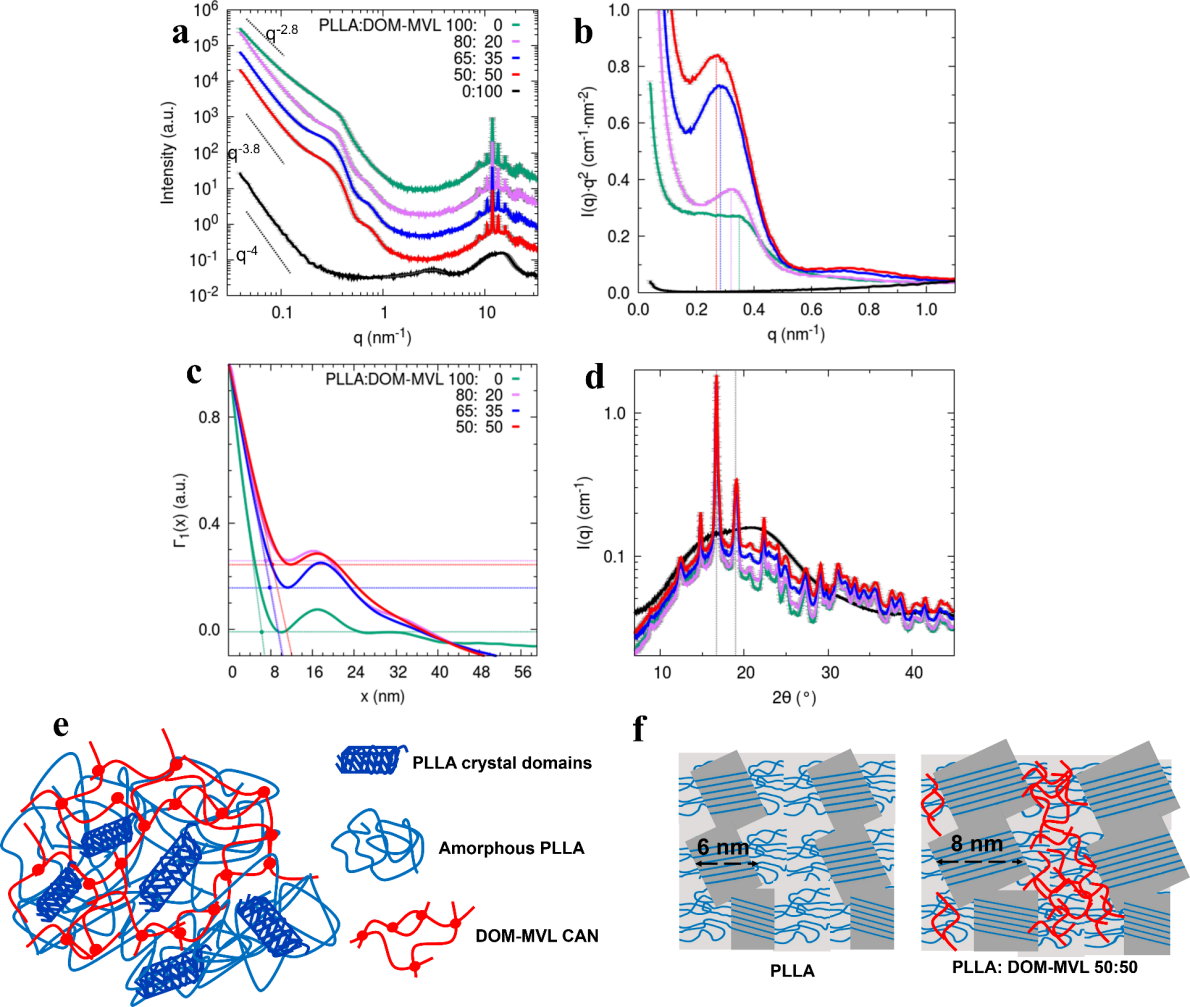
(a) SAXS–WAXS data on the double logarithm scale, with profiles vertically shifted for clarity. Reference power laws are reported for comparison. (b) SAXS data shown as *I*(*q*)·*q* ^2^ in absolute intensity units. The positions of the maxima are highlighted as vertical dotted lines. (c) One-dimensional correlation functions obtained by Fourier inversion of the *I*(*q*)·*q* ^2^ SAXS data, with indication of the extrapolated values of the crystalline lamellar thickness (*lc*). (d) WAXS signals highlighting the positions of the two most intense reflections (200 and 203) as dotted lines. Schematic representation of the phase organization in semi-IPNs from WAXS (e) and SAXS (f) results.

The DOM-MVL CAN, on the other hand, has no signal in the SAXS regime, testifying that the structure is homogeneous in terms of electron density contrast when seen at a scale of 7–30 nm, and only shows broad peaks of an amorphous phase in the WAXS regime (Figure 5a,c). The broad maximum at *q* = 3.1 nm^–1^ should correspond to an approximate spacing of 2 nm, compatible with the molecular architecture of DOM-MVL.

Usually, in pure semicrystalline polymers, it is assumed that the SAXS signal arises from the approximately lamellar alternation of electron density between amorphous regions and crystalline domains having a denser (of the order of 10%) packing of the chains. The position of the broad SAXS peak, *q*_max_, can be used by the Bragg’s law to determine the long period of this repeating structure (*L* = 2π/*q*_max_), corresponding to the sum of the thickness of crystalline and amorphous regions. The plot of *I*(*q*)·*q*2 vs *q* (also called Kratky plot or “Lorentz correction”), shown in Figure 5b, can be used to better visualize both the characteristic broad peak position and the increase in total scattered intensity (the invariant *Q* is the area subtended by this plot and quantifies the amount of nanometer-sized interface weighted by the square of the electron density contrast).

For the pure PLLA sample, the peak is weakly pronounced, and it appears as a “knee” observed before the marked intensity decay at 0.35 nm^–1^, corresponding to a long period of 18 nm. This peak becomes much more pronounced and shifts to lower *q* values when DOM-MVL is added (Figure 5b), documenting an increase of the overall periodic distance from 18 to 23 nm for PLLA:DOM-MVL 65:35 and 50:50. The one-dimensional correlation function (Γ_1_) analysis further highlights how the lamellar thickness profiles are shifted to larger distance values (Figure 5c). The average crystalline lamellar thickness (*lc*) estimated from the linear tangent to the initial Γ_1_ decay is observed to grow from 6 nm for pure PLLA to 8 nm for PLLA:DOM-MVL 50:50. Such increase in the crystal thickness agrees with the progressively increasing Δ*H*_mPLLA_ values measured during the first DSC heating (Table 3), which attest the higher PLLA crystallizability in the presence of uncured resin, as discussed above. It is indeed worth reminding that the SAXS/WAXS measurements were performed on the as-cured samples, characterized by the first DSC heating scan (see Table 3). An additional maximum around 0.7 nm^–1^ emerges for PLLA:DOM-MVL CAN 50:50 and 65:35 (Figure 5b), and since it is found at larger *q* compared to the double of *q*_max_ value, it cannot be ascribed to a second order of the long period, but it could be related to an additional repeating distance of approximately 9 nm ascribed to CAN in the presence of PLLA. The correlation distance in the structure of DOM-MLV could change in the presence of PLLA, and the characteristic spacing increases from 2 to 9 nm.

Intense diffraction peaks are detected in the WAXS region for all samples, testifying to the considerable crystallinity of PLLA that is also maintained in the semi-IPNs (Figure 5d). The position and relative intensity of the second most intense reflection (indexed as 203)^45^ at 13.48 nm^–1^ (19.04° in 2θ), together with the presence of other less intense peaks at higher and lower 2θ values, confirm the prevalence of the more ordered α crystalline form, which is indeed formed above 120 °C, compared to the less packed α’ form, which does not give high SAXS relatively to the amorphous region.^46^

The SAXS and WAXS data collected at increasing temperature up to 90 °C (Figure S8) show an increase of the SAXS signal up to 90 °C, while the WAXS remains approximately constant, in agreement with previous reports.^47^ Such behavior is interpreted as due to the different thermal expansion between amorphous and crystal components with an increase of the electron density contrast between the two phases. The evolution of the PLLA crystalline periodicity is schematized in Figure 5f.

### Effect of PLLA on the Curing of DOM-MVL Resin

In addition to investigating the effects of the DOM-MVL CAN on the crystallization potential of PLLA, the influence of PLLA on the curing efficiency of the resin was also explored.

As a first step, the DOM-MVL CAN and all the semi-IPNs were subjected to solubilization tests in DCM under static conditions to evaluate the solvent resistance of the materials.^48^ After this treatment, all samples were recovered as compact, free-standing films, indicating that solvent exposure did not induce macroscopic fragmentation. The results, summarized in Table 4, show a residue of approximately 78 wt % for the neat DOM-MVL CAN, consistent with the presence of incompletely cured monomers or soluble oligomeric species. On the basis of this value, the expected residue for each semi-IPN composition was calculated and is reported in Table S4. Comparison between the experimental and expected residue values indicates that the PLLA:DOM-MVL 80:20 semi-IPN contains a limited fraction of soluble species. In contrast, the residue measured for the PLLA:DOM-MVL 65:35 system closely matches the expected value, indicating the highest effective curing degree of the resin in this formulation. For the PLLA:DOM-MVL 50:50 semi-IPN, the lower- than-expected residue reveals the presence of a significant amount of unreacted monomers or soluble components, which accounts for the plasticizing effects observed in DSC and DMTA analyses.

**Table 4:** Residue obtained after 72 h immersion in DCM

sample	residue [w/w %]
DOM-MVL CAN	78 ± 5
PLLA:DOM-MVL 80:20	13 ± 3
PLLA:DOM-MVL 65:35	29 ± 3
PLLA:DOM-MVL 50:50	29 ± 8

To further investigate the ability of the DOM-MVL resin to cure in the presence of PLLA, from the extraction experiments under mechanical stirring (72 h, 200 rpm) above-described, the solid residue was recovered. It was approximately 3 ± 1 wt % for the PLLA:DOM-MVL 80:20 system, 21 ± 2 wt % for the 65:35 composition, and 12 ± 3 wt % for the 50:50 semi-IPN. Comparison between these stirred extraction results and the static solubility tests highlights that a fraction of the unreacted or oligomeric material is physically entrapped within the PLLA phase. While this fraction can be efficiently removed under mechanical stirring, it remains partially retained under static conditions, thereby contributing to the solvent resistance of the semi-IPNs.

### Structural Organization of the Two Phases at the Micrometric Scale

The morphology of the as-produced samples and those collected after solubility tests in DCM was examined by using SEM to gather insights into the organization of PLLA and CAN phases at the micrometric scale. For the as-produced DOM-MVL CAN and semi-IPNs, no distinct features were observed, as illustrated in Figure 6a,c,e,g. However, the analysis of the samples postimmersion provided valuable information.

**Figure 6:**
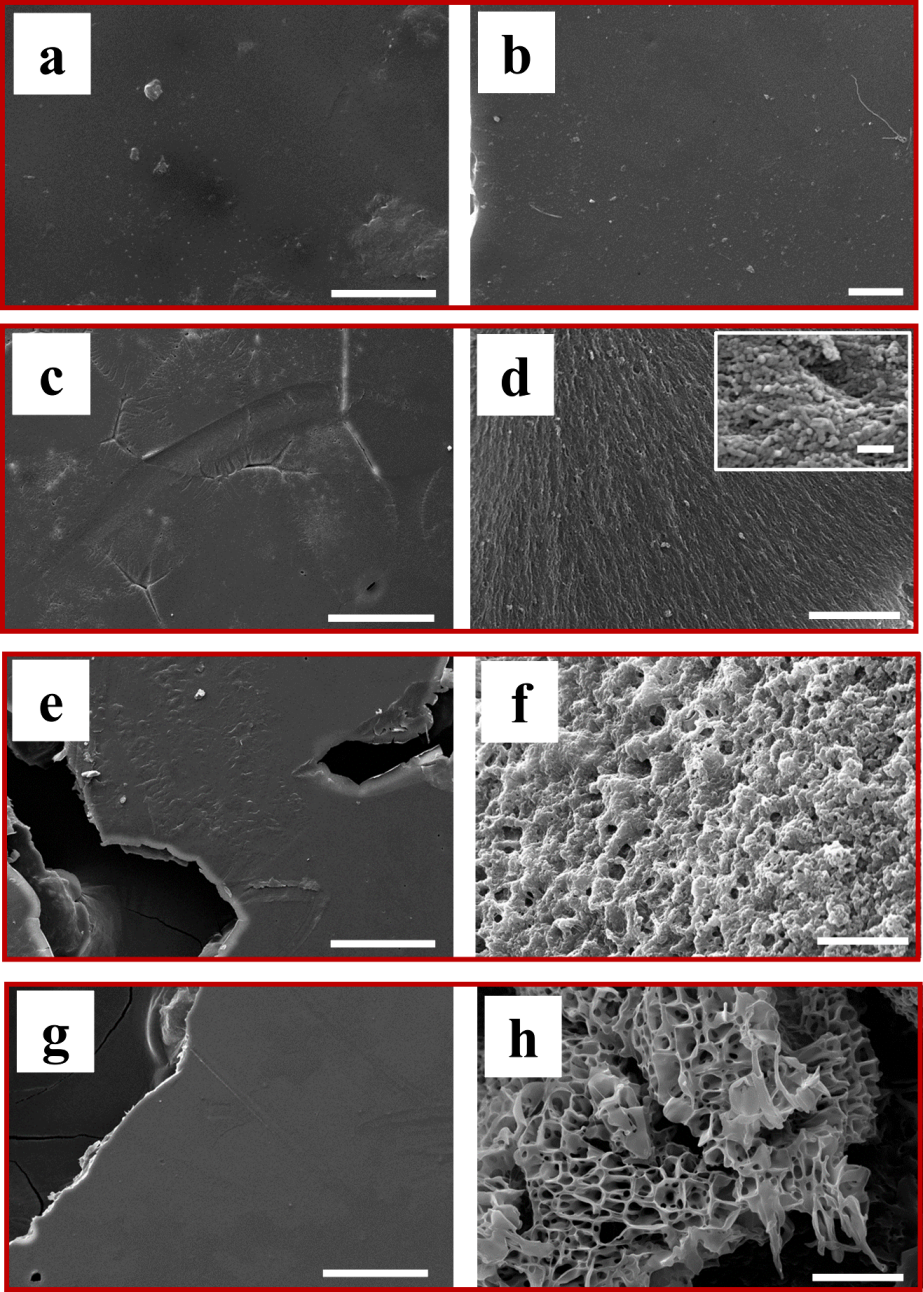
SEM imaging (a, c, e, g) before and (b, d, f, h) after 72 h immersion in DCM of (a, b) DOM-MVL CAN, (c, d) PLLA:DOM-MVL 50:50, (e, f) PLLA:DOM-MVL 65:35, and (g, h) PLLA:DOM-MVL 80:20. Scale bar: 100 μm (a, c, e, g); 10 μm (b, d, f, h); and 1 μm (inset of d).

As expected and in line with a previous study on a similar system,^49^ DOM-MVL CAN displayed a flat and homogeneous morphology even after immersion in DCM (Figure 6b). In contrast, the residue from the semi-IPN 50:50 exhibited a noncompact morphology characterized by aggregated nanometric structures (Figure 6d and inset). This appearance suggests that the removal of PLLA and unreacted monomer or soluble structures from the physical mixture caused a network collapse, making it challenging to obtain information about morphological details. For the residues obtained from PLLA:DOM-MVL = 65:35 (Figure 6f) and PLLA:DOM-MVL = 80:20 (Figure 6h), networks were observed with empty spaces ascribable to regions previously occupied by the PLLA phase. However, it is worth mentioning that during this test, the PLLA phase is removed and the remaining cross-linked structure is swollen by the solvent, with the network junctions pulled apart to accommodate this increase in volume.^50^ Therefore, the volume of the empty regions does not exactly correspond to the dimensions of the PLLA domains in the as-produced systems. Nonetheless, this finding suggests a certain physical miscibility between the two phases and demonstrates the interconnection of the PLLA and CAN phases. However, the results also indicate the presence of regions within the semi-IPNs that are exclusively occupied by the PLLA domains, containing amorphous and crystalline regions, as confirmed by the WAXS results.

### Mechanical Properties of Semi-IPNs

The mechanical properties of PLLA, DOM-MVL CAN, and all the semi-IPNs were investigated by uniaxial tensile tests, and the corresponding stress–strain curves and derived parameters are reported in Figure 7. As expected, neat PLLA exhibits the highest stiffness and strength, whereas the DOM-MVL CAN shows lower modulus and stress at break, consistent with its amorphous, dynamically cross-linked nature. Among the semi-IPNs, the PLLA:DOM-MVL 80:20 composition displays the highest Young’s modulus, reflecting the dominant contribution of the semicrystalline PLLA phase. In contrast, the 50:50 and 65:35 systems exhibit an intermediate mechanical behavior between neat PLLA and the CAN. The stress at break increases systematically with increasing PLLA content, confirming the reinforcing role of the thermoplastic phase. Interestingly, the PLLA:DOM-MVL 65:35 semi-IPN shows the highest strain at break among the semi-IPNs, indicating a more pronounced ductile behavior. This response is consistent with the enhanced phase interpenetration and the network structure inferred from thermal and structural analyses, which promote stress transfer while preserving chain mobility. The highest ductility of PLLA:DOM-MVL 65:35 is also reflected in a Young’s modulus value lower than that of PLLA:DOM-MVL 80:20 and PLLA:DOM-MVL 50:50, confirming the influence of the interconnected and diffused amorphous DOM-MVL regions on the overall response to stress.

**Figure 7:**
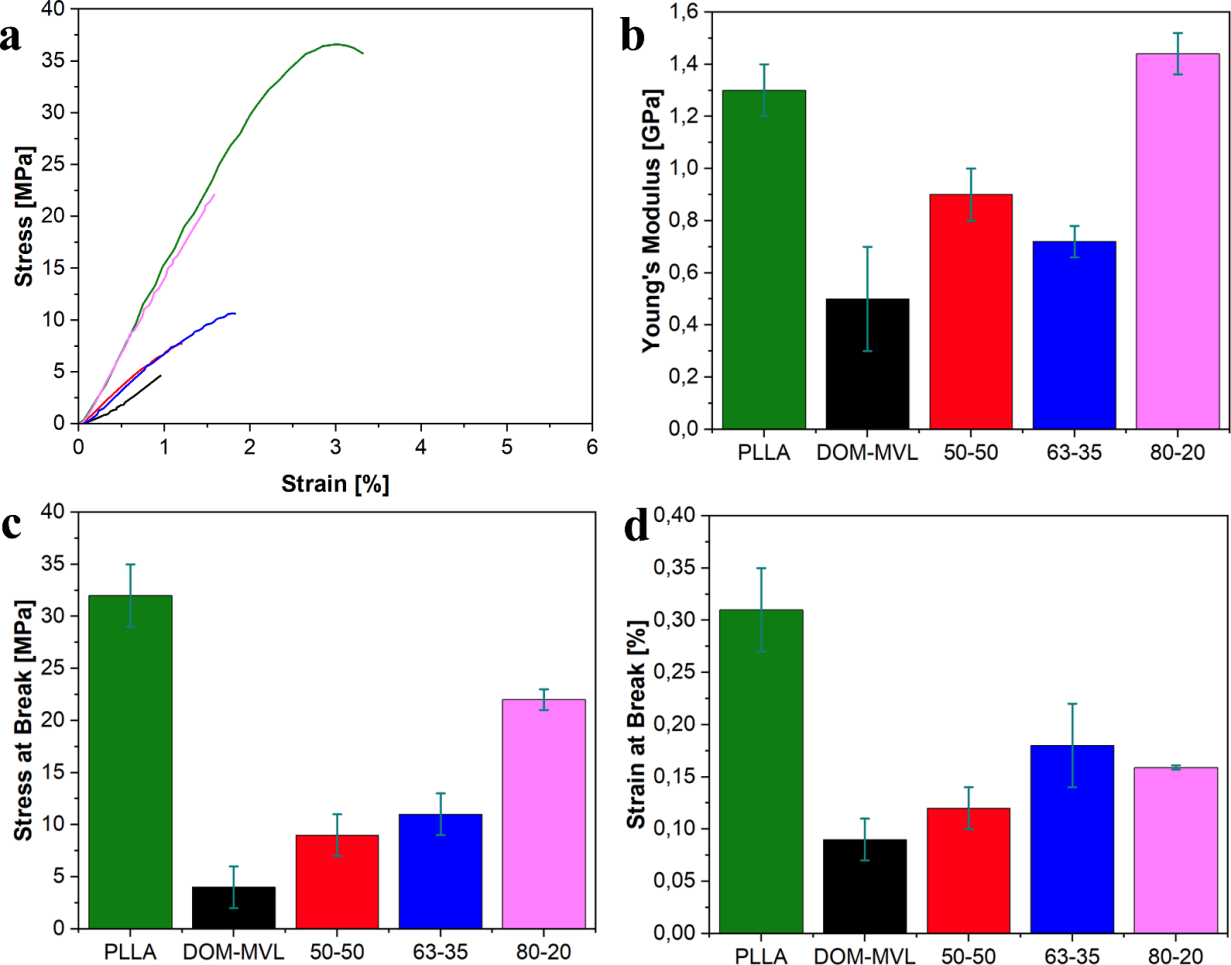
Stress–strain curves (a), Young’s modulus (b), stress at break (c), and strain at break (d) of PLLA (green), PLLA:DOM-MVL 80:20 (red), PLLA:DOM-MVL 65:35 (blue), PLLA:DOM-MVL (50:50), and DOM-MVL (black).

### Reprocessing and Chemical Recycling

The reprocessing of DOM-MVL and semi-IPNs was carried out by cutting the samples into small pieces and subjecting them to hot-pressing as detailed in the experimental section. For all the samples, continuous films were obtained, demonstrating successful reprocessability, as shown in Figure 8a. A slight darkening of the reprocessed semi-IPNs with respect to the original samples was observed with increasing DOM-MVL concentration. While the reprocessing of DOM-MVL relies solely in the metathesis-driven exchange of the imine bonds, the reprocessability of the semi-IPNs benefits from the synergistic effect of the dynamic covalent bonds of the CAN phase and the thermoplastic nature of the PLLA phase. Notably, the applied reprocessing temperature (around 40 °C above the *T*_m_ of PLLA) allows partial melting of the polymer phase. The molten polymer can fill the voids between the CAN domains, promoting the formation of a more compact and homogeneous film.

**Figure 8:**
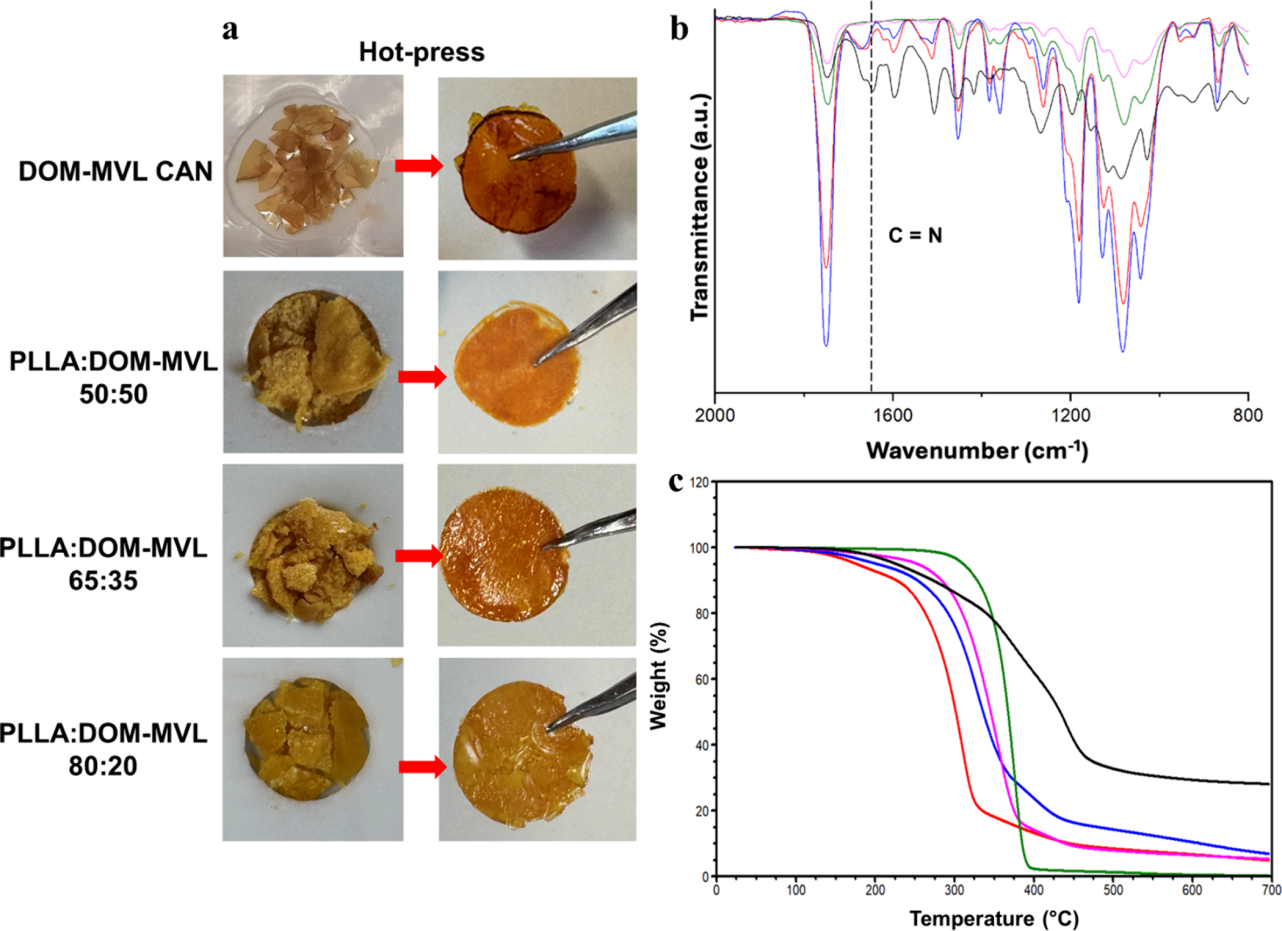
(a) Pictures of the samples before and after the hot-press procedure. Reprocessed samples are held with tweezers. (b) ATR-FTIR spectra and (c) TGA curves of reprocessed thermoset DOM-MVL (black), PLLA:DOM-MVL 50:50 (red), PLLA:DOM-MVL 65:35 (blue), PLLA:DOM-MVL 80:20 (pink), and PLLA (green).

This phenomenon has previously been reported for phase-separated blends of epoxy thermosets and poly­(caprolactone) (PCL), highlighting the possibility of exploiting PCL melting during thermal treatment to enable the formation of thermally mendable polymeric materials.^51,52^ In ATR-FTIR spectra (Figure 8b) after mechanical reprocessing, the imine-related band of DOM-MVL (∼1640 cm^–1^) appears broadened, while in the reprocessed semi-IPNs, it is shifted toward higher wavenumbers (∼1660 cm^–1^). This feature is attributed to overlapping contributions from residual imine bonds and newly formed amide functionalities, possibly generated during hot pressing in the presence of PLLA. While the materials retain macroscopic compactness after reprocessing, these spectral changes suggest that minor side reactions may occur during thermal treatment. From the TGA analysis in Figure 8c and Table S5, it can be observed that thermal reprocessing induces a decrease in the degradation onset (*T*_deg5%_) temperature of the semi-IPNs, but it does not affect the maximum degradation temperature. This result confirms that no significant thermal degradation is induced during the reprocessing.

The effect of mechanical reprocessing on the thermal behavior of the semi-IPNs was further investigated by DSC, and the results are shown in Figure S9 and Table S6. Hot-press reprocessing was performed at 200 °C, a temperature high enough to activate imine bond exchange and to fully melt the PLLA crystalline phase, enabling the formation of compact, free-standing films. A successive fast cooling allowed obtaining amorphous samples. Accordingly, the first DSC heating scan of the reprocessed samples shows a cold crystallization peak. As the PLLA content increases, the position of this peak shifts to higher temperatures, approaching that of the reprocessed neat PLLA, which was analyzed as a reference. The normalized crystallization and melting enthalpies (Δ*H*_cPLLA_ and Δ*H*_mPLLA_) indicate that the overall crystallization ability of PLLA after reprocessing is only moderately influenced by the presence of the DOM-MVL CAN.

Compared to the as-prepared semi-IPNs, the reprocessed materials display an altered crystallization behavior, suggesting changes in phase organization induced by the hot-pressing step.

As a final step, the possibility to separate the two phases of the semi-IPN and use them for the obtainment of two new pristine PLLA films and DOM-MVL CAN has been investigated. The sample subjected to this analysis is the PLLA:DOM-MVL = 65:35 for which a high physical mixing and interconnection between the two phases were demonstrated.

Following the procedure outlined in Figure 9a and described in detail in the experimental section, the PLLA phase was solubilized in DCM, and the resulting solution was solvent cast to form a yellowish film. The yellow coloration was attributed to the presence of residual unreacted DOM-MVL resin or oligomeric structure present with a concentration of 18 wt % with respect to the total weight of the extract, as confirmed by ATR-FTIR (Figure 9b), ^1^H NMR (Figure S6), and TGA analyses (Figure S7 and Table S3). Subsequent immersion in acetone effectively removed the residual monomer, yielding recovered PLLA films, as verified by both ATR-FTIR (Figure 9b) and TGA (Figure 9d) analyses. However, although the ATR-FTIR and TGA analyses indicate the preservation of the main spectroscopic and thermal features of PLLA after recovery, a detailed investigation of possible molecular weight changes induced by aminolysis reactions was beyond the scope of the present study.

**Figure 9:**
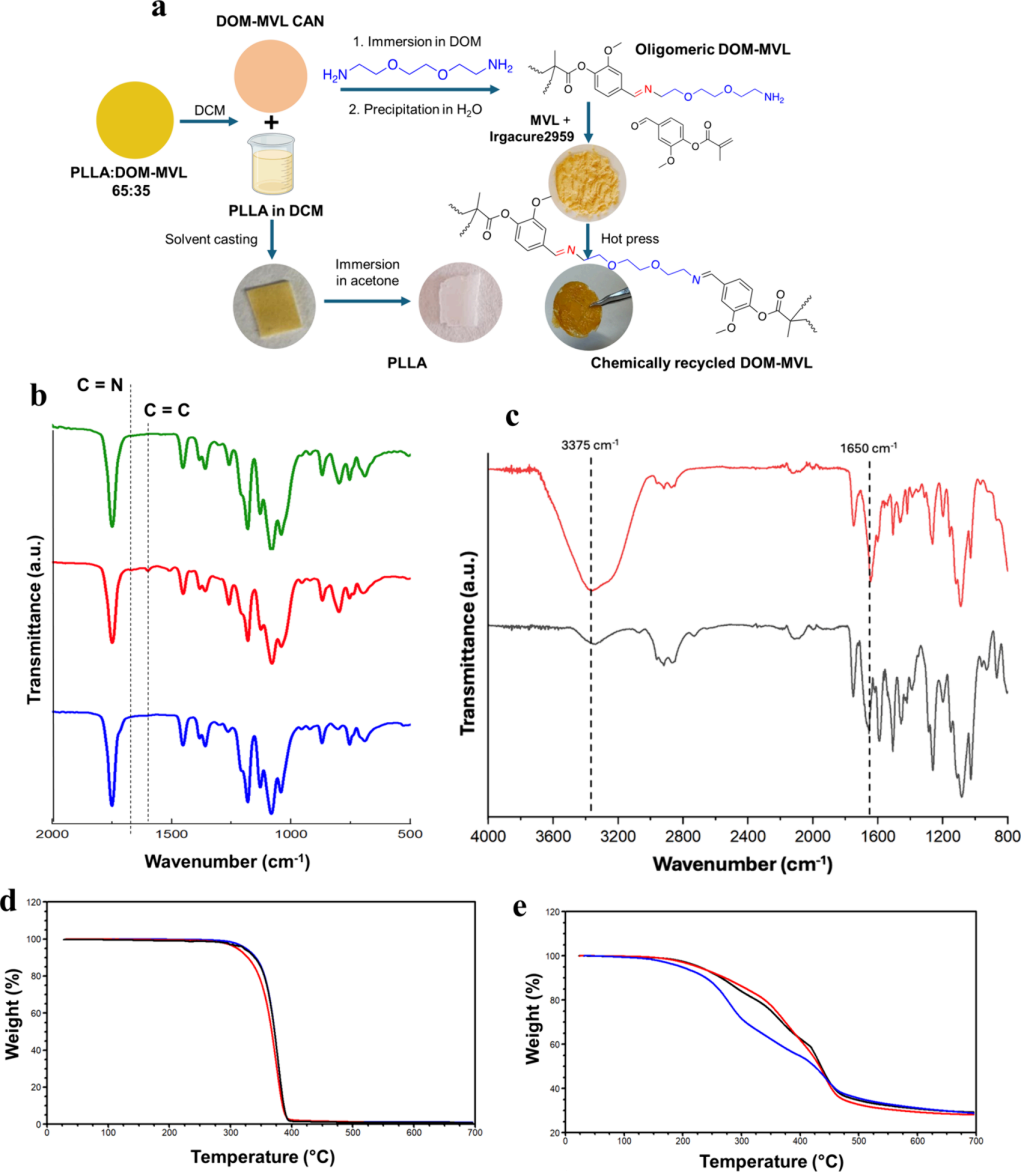
(a) Scheme of the procedure for the chemical recycling of PLLA:DOM-MVL films. (b) ATR-FTIR spectra of the PLLA film (green), chemically recycled PLLA film recovered after the separation of the two components of the PLLA:DOM-MVL film (red), and chemically recycled PLLA film after washing in acetone (blue). (c) ATR-FTIR spectra of the oligomeric DOM-MVL (red) and chemically recycled thermoset (black). (d) TGA curves of the as-produced PLLA (black), reprocessed PLLA (red), and chemically recycled PLLA (blue). (e) TGA curves of the as-produced thermoset (black), reprocessed thermoset (red), and chemically recycled thermoset (blue).

The remaining solid fraction, composed of DOM-MVL CAN, was chemically recycled following previously established protocols.^34^ Briefly, the DOM-MVL was immersed in excess DOM to activate the transamination pathway, resulting in the formation of branched oligomers bearing terminal amine groups. These oligomers were recovered and hot-pressed with MVL and a photoinitiator, promoting the formation of new imine bonds in the solid state and subsequent thermal curing, resulting in chemically recycled DOM-MVL CAN. The recycled DOM-MVL CAN exhibited a chemical structure comparable to the original but with slightly reduced thermal stability (Figure 9e), likely due to the partial cleavage of nondynamic covalent bonds during the recycling process.

## Conclusions

PLLA/CAN semi-IPNs were prepared by curing the vitrimeric resin in the presence of PLLA. Characterization results demonstrated physical mixing and interconnection between the two phases. Increasing the resin content from 35 to 50 wt % resulted in the presence of isolated resin monomers in the PLLA phase, acting as plasticizers. This plasticization effect broadened the crystallization window and enhanced the crystallization potential of the PLLA. Conversely, a lower resin content (20 wt %) did not enable complete curing, likely due to the high dispersion of resin within the PLLA matrix. The PLLA:DOM-MVL 65:35 composition exhibited the highest curing degree of the CAN phase and the most effective physical interpenetration between the two components, as evidenced by the marked reduction in the crystallization potential of PLLA.

SEM imaging and SAXS/WAXS analyses revealed micro- and nanoscale structural organization, respectively. Notably, SAXS results indicated that the DOM-MVL CAN phase produces in PLLA an enhancement of the crystalline domain thickness from 6 nm for pure PLLA to 8 nm for PLLA:DOM-MVL 50:50.

On the basis of overall experimental results, the resulting materials can therefore be described as PLLA/CAN semi-IPNs containing variable amounts of entrapped uncured or oligomeric resin species.

The successful reprocessing of the semi-IPNs was enabled by the metathesis-driven exchange of imine bonds within the CAN phase and the thermoplastic nature of PLLA. The partial retention of imine linkages in the reprocessed semi-IPNs confirms that dynamic covalent bond exchange occurred during hot pressing, while the absence of significant thermal degradation underscores the compatibility of the process with the material properties. Moreover, the component separation was achieved by simple immersion of the semi-IPN in DCM. This procedure enabled the recovery of PLLA films, preserving the characteristic thermal and spectroscopic features of neat PLLA, together with a porous CAN network that was subsequently chemically recycled into a new vitrimer via imine transimination.

In conclusion, this work presents a comprehensive multiscale characterization approach to investigate the structural organization of a semicrystalline thermoplastic and a vitrimer within semi-IPN systems. The promising results in terms of reprocessability and chemical recyclability pave the way for the development of next-generation multicomponent plastic materials, for which recycling and component recovery remain persistent challenges.

## Supplementary Material



## Data Availability

The raw experimental data supporting the findings of this study, including DSC, DMTA, TGA, SAXS/WAXS, ATR-FTIR spectra, and mechanical testing data, are available in the [AMS Acta] repository. The data set can be accessed at [https://amsacta.unibo.it/id/eprint/8384/].

## Notes

^1^H NMR spectra of methacrylated vanillin and DOM-MVL (Figure S1); ATR-FTIR spectra of vanillin, methacrylated vanillin, and Schiff-base resin (Figure S2); ATR-FTIR spectra of DOM-MVL after different curing procedures (Figure S3); DSC data obtained from the first heating scan on DOM-MVL when subjected to different curing procedures (Figure S3 and Table S1); first calorimetric heating scan of UV-cured DOM-MVL, PLLA:DOM-MVL 50:50, PLLA:DOM-MVL 65:35, PLLA:DOM-MVL 80:20, and PLLA (Figure S4); DSC data from the first heating scan performed on noncured systems (Table S2); schematic representation of the organization of PLLA and DOM-MVL phases in semi-IPNs (Figure S5); ^1^H NMR spectra of solvent-cast films obtained from the extracts of all the samples (Figure S6); TGA curves and residues of solvent-cast films obtained from the extracts of all the samples (Figure S7 and Tables S3); expected residues after solubility tests (Table S4); SAXS and WAXS data as a function of temperature (Figure S8); TGA analysis of reprocessed samples (Table S5); and DSC curves and data from the first heating scan performed on reprocessed samples (Figure S9 and Table S6) (PDF)
