# Supplementary material for: Phase Organization and Circularity in PLLA/Vitrimer Semi-interpenetrating Polymer Networks
Source: Macromolecules. 2026 Jun 16;59(13):7862–78. doi: 10.1021/acs.macromol.6c00678 (PMC13374509; doi:10.1021/acs.macromol.6c00678)
Supplement: Supplementary file 1 [file ma6c00678_si_001.pdf]

Supporting Information

Phase Organization and Circularity in PLLA/Vitrimer Semi-Interpenetrating Polymer Networks

Luigi Gamberini, Alessandra Del Giudice, Lazaros Papadopoulos, Maria Cristina Righetti, Minna Hakkarainen, Luciano Galantini, Anna Liguori*, Maria Letizia Focarete

*anna.liguori@unibo.it

Synthesis of DOM-MVL

^{1}H-NMR peaks of MVL: ^{1}H NMR (600 MHz, $CDCl_3$- d1): δ ppm 9.96 (s, 1H, –CHO), 7.51 (d, 1H, Ar), 7.49 (s, 1H, Ar), 7.25 (d, 1H), 6.39 (s, 1H, -C=CH2), 5.81 (t, 1H, -C=CH_2), 3.91 (s, 3H, –OCH_3), 2.07 (s, 3H, -C-CH_3).

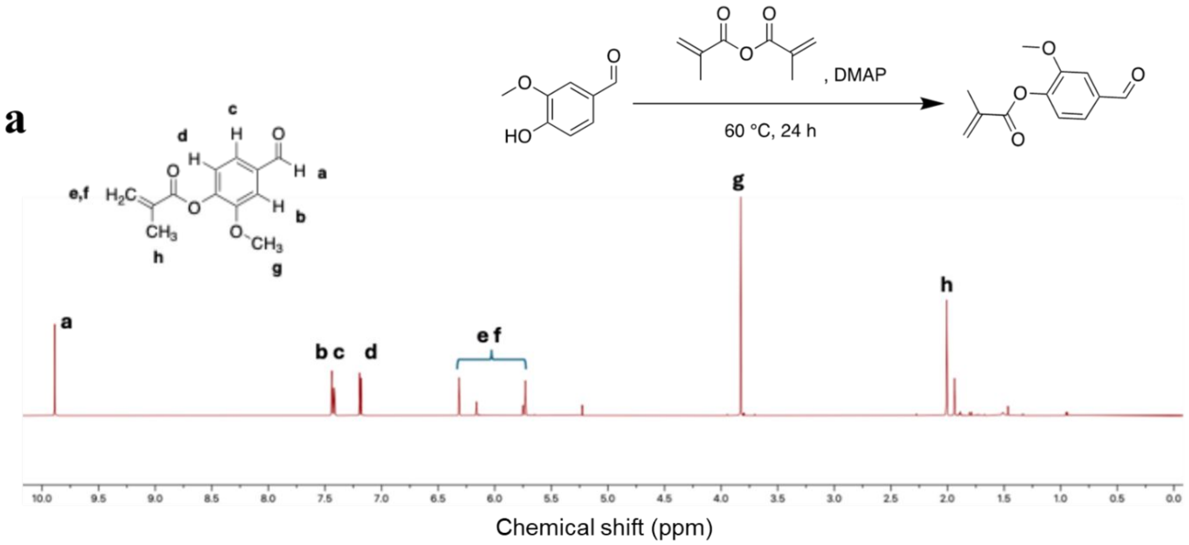

^{1}H-NMR peaks of DOM-MVL: ^{1}H NMR (600 MHz, CDCl3- d1): 8.26 (s, 2H, –HCN-), 7.50 (d, 2H, Ar), 7.21 dd, 2H, Ar), 7.12 (m, 2H, Ar), 6.38 (s, 2H, -C=CH2), 5.79 (t, 2H, -C=CH2), 3,88 (s, 6H, –OCH3), 3,79 (s, 8H, –H2COCH2CH2OCH2-), 3,66 (m, 4H, -CN-CH2-), 2.09 (s, 3H, -C-CH3).

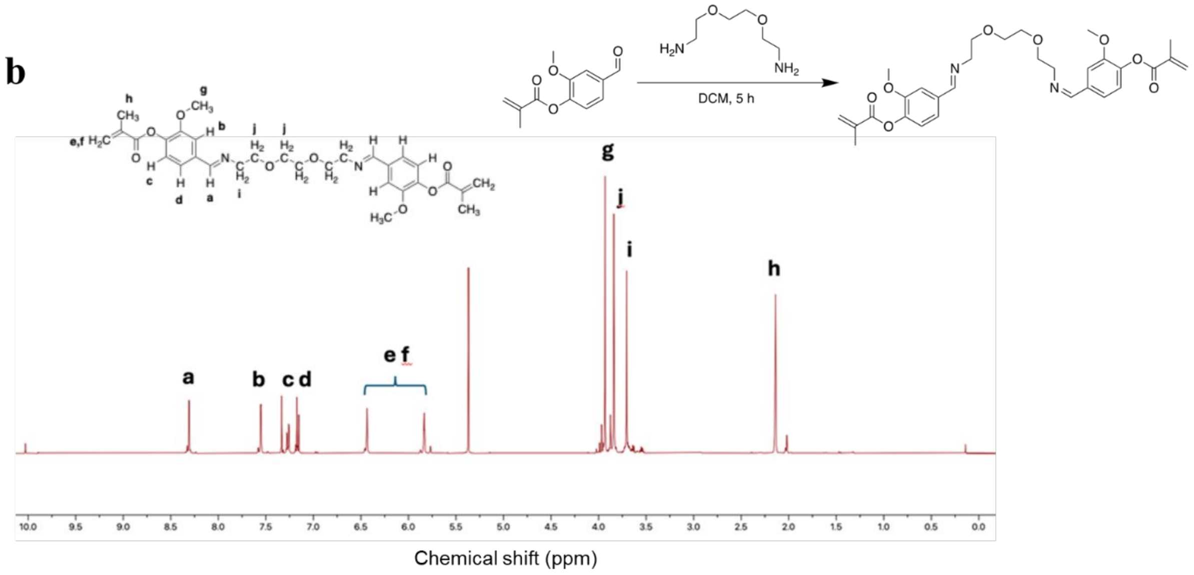

Figure S1. Schemes of reaction and ^{1}H-NMR spectra for (a) methacrylated vanillin (MVL) and (b) Schiff-base resin (DOM-MVL).

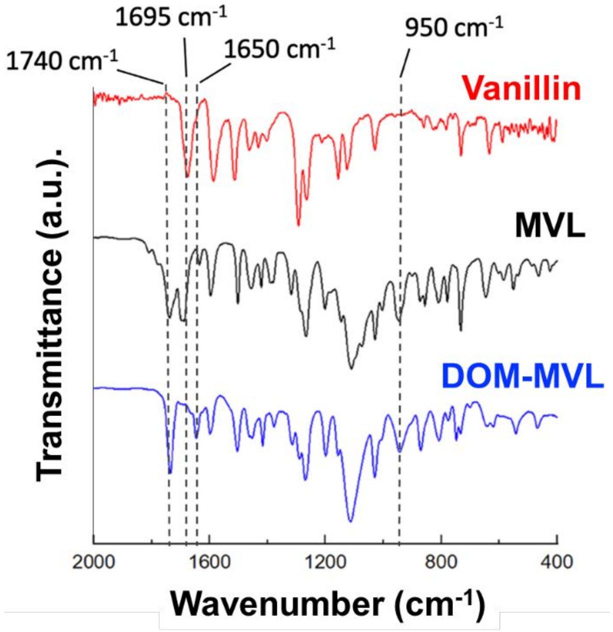

Figure S2. ATR-FTIR spectra of vanillin, methacrylated vanillin (MVL) and Schiff-base resin (DOM-MVL)

Optimization of the procedure for the curing of DOM-MVL CAN

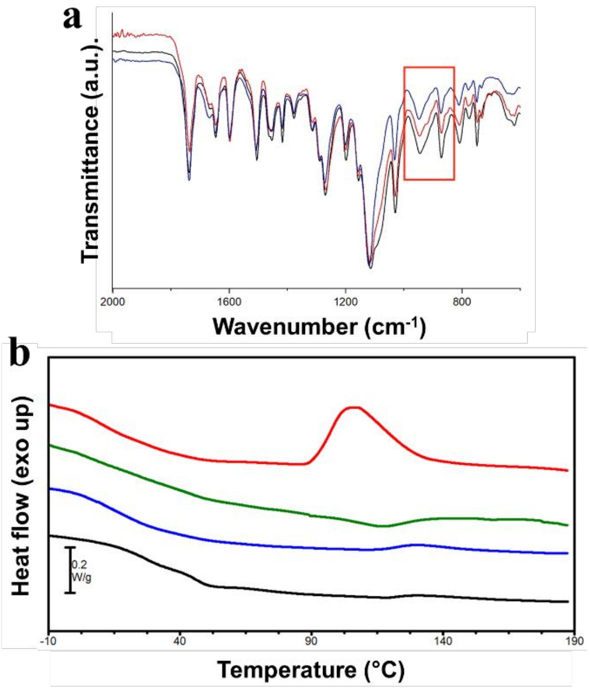

Figure S3. (a) ATR-FTIR spectra of DOM-MVL after UV curing for 10 min (black), 15 min (red) and 1 h (blue); no thermal treatment is performed. (b) First calorimetric heating scan on DOM-MVL subjected to UV curing for 1 h (red), UV curing for 1 h + thermal treatment at 110 °C for 15 min (green), UV curing for 1 h + thermal treatment at 130 °C for 15 min (blue), UV curing for 1 h + thermal treatment at 130 °C for 1.5 h (black)

Table S1. DSC data obtained from the first heating scan on DOM-MVL when subjected to different curing procedures.

**Curing procedure**	**Tg [°C]**	**ΔC_p [J/g °C]**	**T_{curing} [°C]**	**ΔH_{curing} [J/g]**
UV curing for 1 h	17	0.62	107	19
UV curing for 1 h + thermal treatment at 110°C for 15 min	20	0.74	145	5
UV curing for 1 h + thermal treatment at 130°C for 15 min	20	0.73	131	2
UV curing for 1 h + thermal treatment at 130°C for 1.5 h	31	0.58	131	1

DSC analysis of PLLA:DOM-MVL after the UV curing (no thermal treatment)

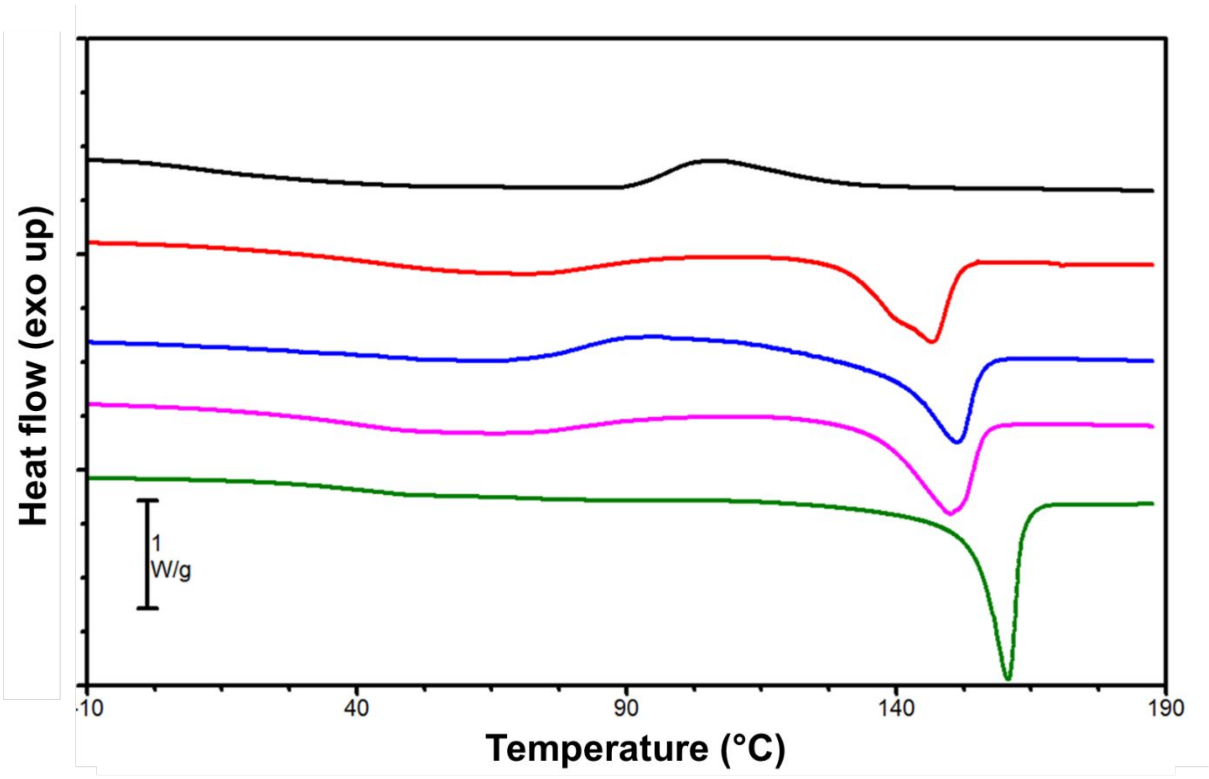

Figure S4. First calorimetric heating scan of UV cured DOM-MVL (black), PLLA:DOM-MVL 50:50 (red), PLLA:DOM-MVL 65:35 (blue), PLLA:DOM-MVL 80:20 (pink), PLLA (green).

Characterizations of PLLA:DOM-MVL before the curing procedure

Table S2. Tg and ΔCp from the first heating scan performed on non-cured systems.

**Sample**	**$T_{g\ resin}$ [°C]**	**$\Delta C_{p\ resin}$ [J/g °C]**	**$T_{g\ PLLA}$ [°C]**	**$\Delta C_{p\ resin}$ [J/g °C]**
DOM-MVL CAN	-29	0.58	-	-
PLLA:DOM-MVL 50:50	-29	0.23	46	0.17
PLLA:DOM-MVL 65:35	-25	0.10	48	0.25
PLLA:DOM-MVL 80:20	Difficult to measure	Difficult to measure	45	0.25
PLLA	-	-	54	0.42

Organization of PLLA and DOM-MVL phases in semi-IPNs

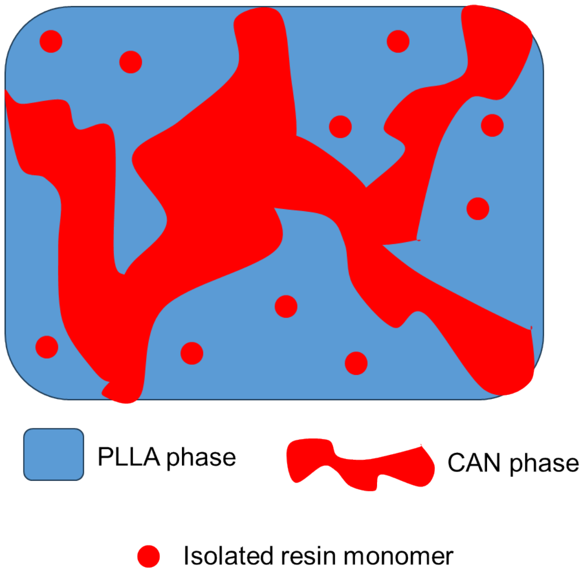

Figure S5. Schematic representation of co-continuous PLLA and CAN phases. The dimension of the CAN phase and the amount of the isolated resin monomer depend on the semi-IPN composition.

Characterizations of PLLA:DOM-MVL after the curing procedure

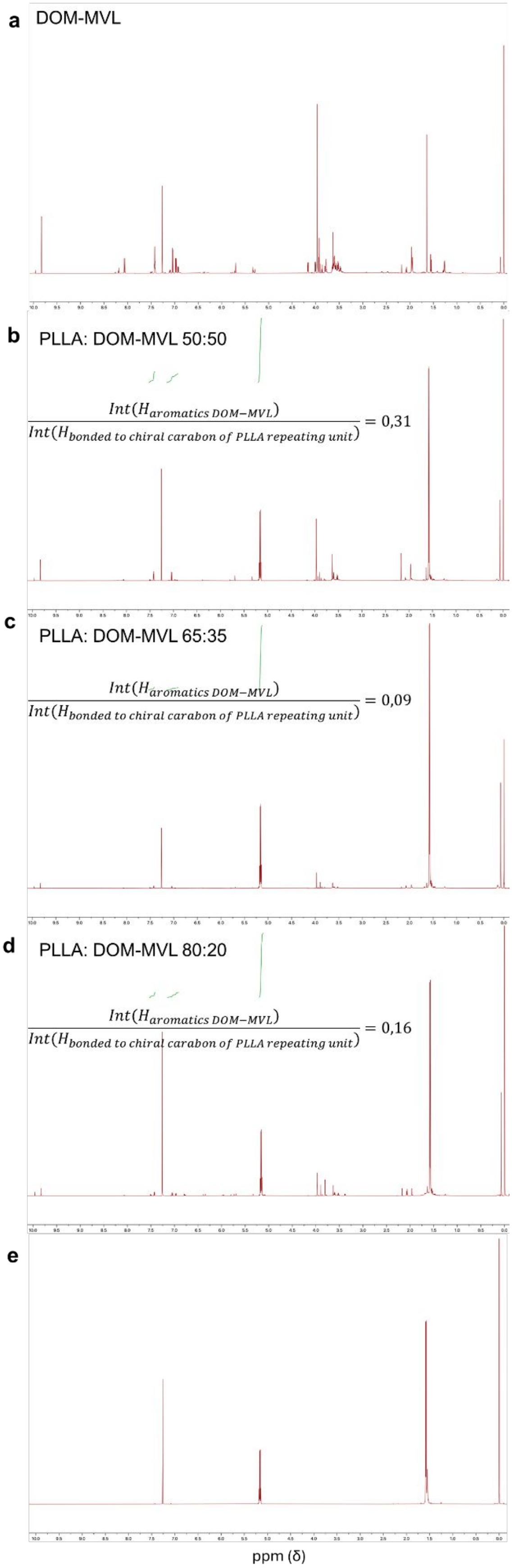

Figure S6. ^{1}H NMR spectra of the solvent-cast extracts obtained after DCM extraction of PLLA:DOM-MVL 80:20, PLLA:DOM-MVL 65:35, and PLLA:DOM-MVL 50:50 semi-IPNs. The spectra are compared with those of solvent-cast DOM-MVL extract and of PLLA.

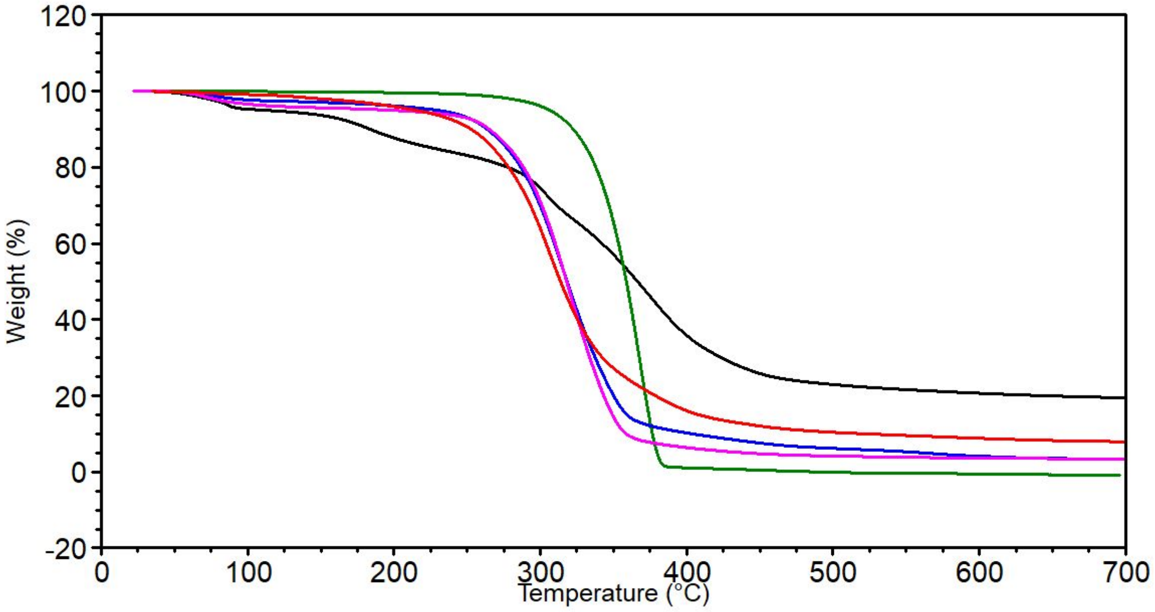

Figure S7. TGA curves of solvent-cast films obtained from the extracts of PLLA (green), PLLA:DOM-MVL 80:20 (pink), PLLA:DOM-MVL 65:35 (blue), PLLA:DOM-MVL 50:50 (red), DOM-MVL (black).

Table S3. Residue at 700 °C determined by TGA for solvent-cast films obtained from DCM extracts after the extraction tests, used to estimate the fraction of unreacted or oligomeric DOM-MVL species in the extracts.

**Sample**	**Residue [w/w%] due to unreacted resins/oligomeric structures in the extracts at 700°C [w/w%]**	**Calculated residue [w/w%] in the extracts taking into account the residue of the pure resin at 700°C**
DOM-MVL CAN	20	-
PLLA:DOM-MVL 80:20	3.6	18
PLLA:DOM-MVL 65:35	3.6	18
PLLA:DOM-MVL 50:50	8	40

Table S4. Expected residue calculated referring to the residues' average values, obtained in static conditions, and considering the residue of thermoset DOM-MVL in DCM.

**Sample**	**Expected residue [w/w%]**
DOM-MVL CAN	-
PLLA:DOM-MVL 80:20	16
PLLA:DOM-MVL 65:35	27
PLLA:DOM-MVL 50:50	39

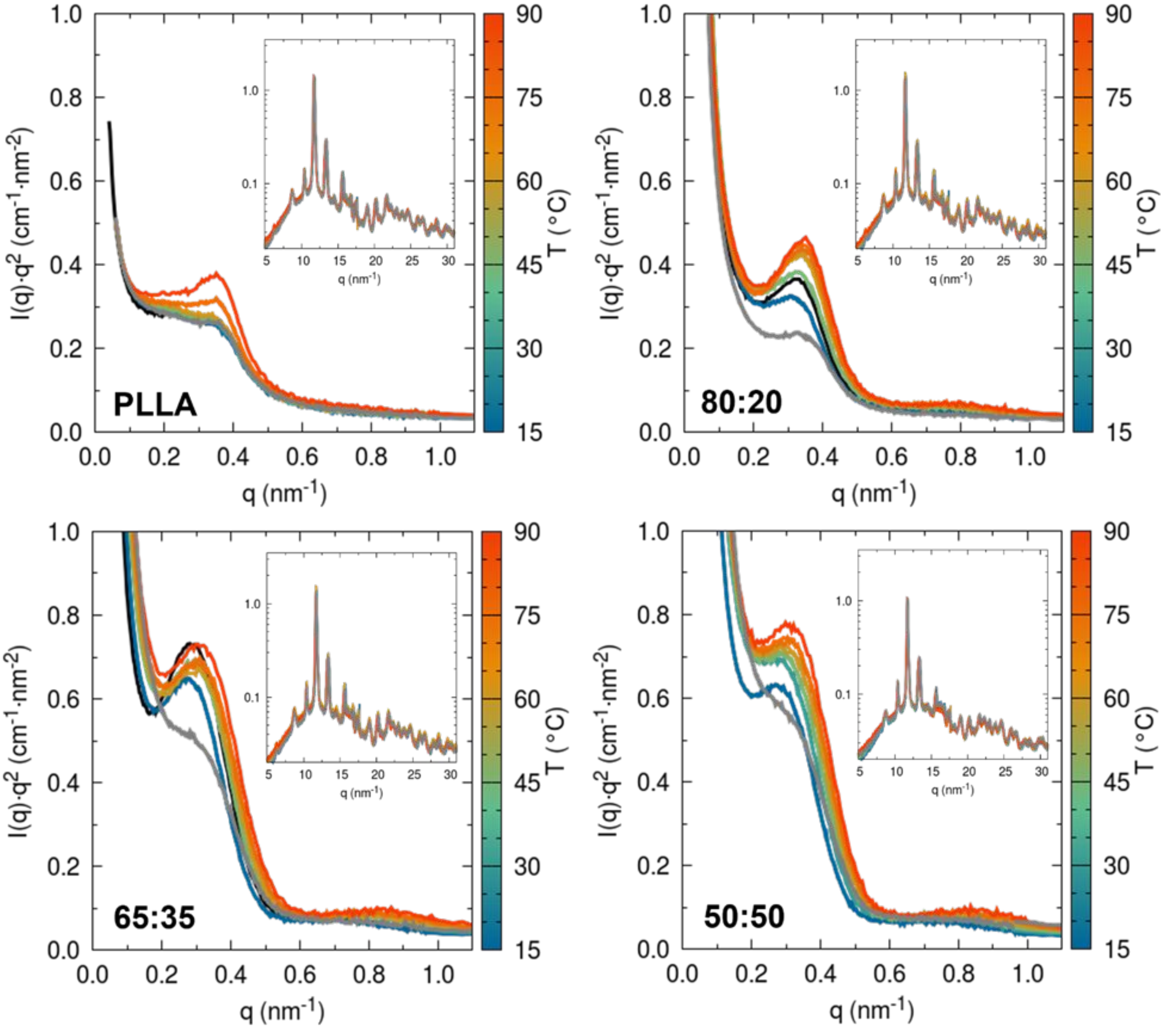

Figure S8. SAXS and WAXS data as a function of temperature (15°C blue, 30°C cyan, 45°C green, 60°C brown, 75°C orang, 90°C red, after quenching to 20°C grey). The data collected at room temperature on the starting samples are reported as black lines. SAXS data are shown as I(q)·q2 (“Kratky plot” or “Lorentz correction”) to emphasise the position and intensity of the broad peak related to the long spacing of the amorphous-crystalline electron density contrast. WAXS data are reported with intensity in Log scale in the insets. (a) PLLA, (b) PLLA:DOM-MVL = 80:20, (c) PLLA:DOM-MVL = 65:35, (d) PLLA:DOM-MVL = 50:50.

Characterizations of reprocessed samples

Table S5. Thermogravimetric data from TGA curves of reprocessed samples

Sample	$T_{deg5\%}$ [°C]	$T_{v\ degmax}$ [°C]	Residue at 700°C [w/w%]
DOM-MVL CAN	226	376, 440	28
PLLA:DOM-MVL 50:50	174	309	5

PLLA:DOM-MVL 65:35	201	327	7
PLLA:DOM-MVL 80:20	253	357	5

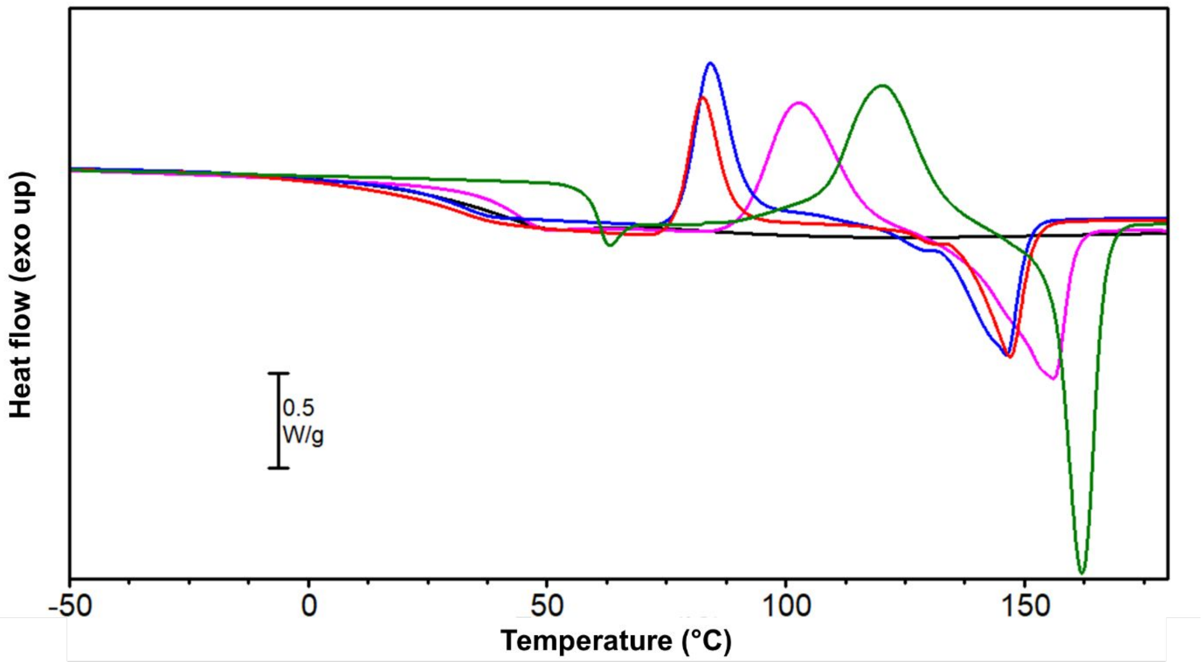

Figure S9. DSC curves of first calorimetric heating scan of PLLA (green), PLLA:DOM-MVL = 80:20 (pink), PLLA:DOM-MVL = 65:35 (blue), PLLA:DOM-MVL = 50:50 (red), DOM-MVL CAN (black).

Table S6. DSC data from the first heating scan of reprocessed samples.

Sample	T_c [°C]	ΔH_c [J/g]	ΔH_{cPLLA} [J/g]	T_m [°C]	ΔH_m [J/g]	ΔH_{mPLLA} [J/g]
DOM-MVL CAN	-	-	-	-	-	-
PLLA:DOM-MVL 50:50	83	16	32	147	24	48
PLLA:DOM-MVL 65:35	84	23	35	146	31	48
PLLA:DOM-MVL 80:20	103	33	41	156	36	45
PLLA	120	42	42	156	45	45
